# Serum profile changes in postpartum women with a history of childhood maltreatment: a combined metabolite and lipid fingerprinting study

**DOI:** 10.1038/s41598-018-21763-6

**Published:** 2018-02-22

**Authors:** Alexandra M Koenig, Alexander Karabatsiakis, Thomas Stoll, Sarah Wilker, Thomas Hennessy, Michelle M Hill, Iris-Tatjana Kolassa

**Affiliations:** 1 Clinical & Biological Psychology, Institute of Psychology and Education, Ulm University, Albert-Einstein-Allee 47, 89081 Ulm, Germany; 2 The University of Queensland Diamantina Institute, The University of Queensland, Translational Research Institute, 37 Kent Street, Woolloongabba, QLD 4102 Australia; 3 Agilent Technologies, 679 Springvale Road, Mulgrave, VIC 3170 Australia

## Abstract

Childhood maltreatment (CM) can increase the risk of adverse health consequences in adulthood. A deeper insight in underlying biological pathways would be of high clinical relevance for early detection and intervention. The untargeted investigation of all detectable metabolites and lipids in biological samples represents a promising new avenue to identify so far unknown biological pathways associated with CM. Using an untargeted approach, liquid chromatography-mass spectrometry (LC-MS) was performed on peripheral blood serum samples collected three months postpartum from 105 women with varying degrees of CM exposure. Comprehensive univariate and multivariate statistical analyses consistently identified eight biomarker candidates putatively belonging to antioxidant-, lipid-, and endocannabinoid-associated pathways, which differentiated between women with and without CM. Classification algorithms allowed for clear prediction of the CM status with high accuracy scores (~80–90%). Similar results were obtained when excluding all women with a lifetime psychiatric diagnosis. In order to confirm the identities of these promising biomarker candidates, LC-MS/MS analysis was applied, confirming one of the metabolites as bilirubin IXa, a potent antioxidant with immunomodulatory properties. In sum, our results suggest novel pathways that could explain long-term effects of CM on health and disease by influencing biological patterns associated with energy metabolism, inflammation, and oxidative stress.

## Introduction

Cumulative early life stress increases the risk for long-term adverse consequences on mental and physical health and thus represents a central societal challenge. Individuals with experiences of childhood maltreatment (CM), including abuse and neglect, report a poorer life quality and overall health^1^ and are at higher risk to develop psychopathologies such as major depressive disorder (MDD) or posttraumatic stress disorder (PTSD). Over one-half of MDD and PTSD cases are potentially attributable to self-reported CM^2^. A deeper understanding of the underlying biological mechanisms, which determine health and disease outcomes following CM experiences, is of highest clinical interest^3^. To enable preventive interventions for individuals at high risk for adverse long-term consequences of chronic and traumatic stress associated with CM, the identification of biological entities in the form of a “biomolecular marker panel” is needed.

Several recent genome-wide epigenetic studies investigated methylation changes in DNA prepared from blood^4,5^, buccal epithelial samples^6^, hippocampal tissue^7^, or saliva^8^ from participants with CM and matched controls. These studies all showed wide-spread epigenetic changes in CM participants, impacting diverse pathways relevant to health risks associated with CM, such as neuronal development and organismal growth^6^, cellular/neuronal plasticity^7^, transcriptional regulation/development^4^, various cancers, signaling and inflammatory pathways^8^. While these studies reveal potential epigenetic mechanisms of increased health risks following CM exposure, there are limited reports on the physiological and biochemical processes associated with CM.

The majority of previous research on the biochemical processes in CM primarily employed hypothesis-driven pathway investigations^9^, thereby neglecting potentially complex risk and resilience pathways related to CM. The individual biochemical fingerprint represents the complex interplay of the genetic make-up, and environmentally shaped gene and protein expression of a person^10^, and provides the highest resolution to assess metabolic changes associated with pathophysiological conditions at a certain point in time. For this reason, *metabolomics* might be able to contribute to the elucidation of gene-environment interactions^11^ leading to a better understanding of the biological and psychosomatic consequences of early life stress.

*Metabolomics* is the science of the human metabolome comprising the total of small hydrophilic (metabolites), amphiphilic (fatty acids), and lipophilic (lipids) bioactive molecules present in cells, tissues, organs, and biological fluids, which display direct signatures of biochemical activity^12^. Untargeted metabolomics, also termed *metabolite fingerprinting*, encompasses the relative quantification of all detectable metabolites in a given biological sample by mass spectrometry.

Traditionally, human metabolomics research addressed somatic conditions such as drug response^10^, diseases of the central nervous system (e.g.^13,14^), cancer^15^, or aging^16^. Only a limited number of studies has been conducted that applied *untargeted metabolomics* in the field of psychiatry, in particular on schizophrenia and MDD (see refs^11,17^ for an overview). A few recent studies investigated the metabolite profiles of autism^18^, bipolar disorder^19^, PTSD^20^, and smoking behavior^21^. To the best of our knowledge, the impact of early life stress on the human metabolome has been reported in one study^22^. Ding and colleagues (2014) discovered a distinct plasma diagnostic metabolite marker panel in depressed patients with a history of early life stress compared to depressive patients without such experiences and healthy individuals. However, there are no published studies on the metabolomic signature of CM experiences in healthy adult subjects.

In a cohort of postpartum women, this study applied a biomarker discovery approach employing untargeted mass spectrometry-based metabolomics for both metabolites and lipids to unravel novel serum biomarkers for CM.

## Results

A subset of 105 serum samples from the “My Childhood – Your Childhood” project was selected for the current analysis (see Supplementary Methods for more details). According to the cut-off criteria of the *Childhood Trauma Questionnaire* (CTQ)^23,24^, we divided the samples into two groups (see Table 1 for descriptive characteristics): a control group including 46 women without CM experiences (CM−) and a group with 59 women reporting a history of at least mild up to severe maltreatment in childhood (CM+). The metabolite and lipid fractions were extracted from serum samples and analyzed by a high-resolution accurate-mass QTOF UHPLC/MS system, which is increasingly considered as the reference platform for metabolomics^25^. Accurate mass data which matched to the METLIN Metabolomics Database (The Scripps Research Institute, USA) was filtered for known endogenous metabolites and lipids, and then by detection in a minimum 50% of samples in each group. Ultimately, 398 compounds (ions with a specific retention time and mass-to-charge ratio) were selected for extensive statistical analysis to discover a biomarker signature that can distinguish CM+ from CM− (see Supplementary Table S1 for a list in alphabetical order). In addition, compound identification using tandem mass spectrometry (LC-MS/MS) was applied (see Supplementary Methods).

**Table 1:** Demographic and clinical characteristics of women with and without childhood maltreatment history.

		Whole study cohort (*N* = 105)	CM group (*N* = 59)	Control group (*N* = 46)	Statistic^g^
Age in years, *M* ± *SD*		32.89 ± 3.99	33.14 ± 3.88	32.57 ± 4.15	*t*(103) = −0.73, *p* = 0.47
German origin: yes, *n* (%)		91 (87)	49 (83)	42 (91)	*p* = 0.26
Married: yes, *n* (%)^a^		83 (79)	48 (82)	35 (76)	*p* = 0.53
Academic education: yes, *n* (%)		73 (69)	38 (64)	35 (76)	*p* = 0.19
Living index (rooms/persons ratio)^a^, *M* ± *SD*		1.28 ± 0.47	1.25 ± 0.52	1.31 ± 0.39	*W* = 1566, *p* = 0.12
Number of children^a^, *M* ± *SD*		1.62 ± 0.79	1.66 ± 0.76	1.57 ± 0.83	*W* = 1202*, p* = 0.34
Body mass index^a^, *M* ± *SD*		23.69 ± 2.98	23.92 ± 3.26	23.41 ± 2.61	*t*(102) = −0.85, *p* = 0.40
Chronical diseases, *n* (%)^a,b^		33 (31)	20 (34)	13 (28)	*p* = 0.53
	Thyroid disease, *n* (%)	21 (20)	11 (19)	10 (22)	*p* = 0.81
	Asthma, *n* (%)	6 (6)	4 (7)	2 (4)	*p* = 0.69
Taken medication at the day of blood sampling: yes, *n* (%)^b^		38 (36)	22 (37)	16 (35)	*p* = 0.84
	Levothyroxin, *n* (%)	21 (20)	11 (19)	10 (22)	*p* = 0.81
	Nutritional supplement, *n* (%)	10 (10)	7 (12)	3 (7)	*p* = 0.51
	Contraceptive, *n* (%)	7 (7)	1 (2)	6 (13)	*p* = 0.04
	Analgesic, *n* (%)	4 (4)	3 (5)	1 (2)	*p* = 0.63
	Asthma inhaler, *n* (%)	2 (2)	2 (3)	0 (0)	*p* = 0.51
Diagnosis of psychiatric disorder lifetime (self-reported): yes, *n* (%)^c,d^		11 (10)	11 (19)	0 (0)	*p* < 0.001
Diagnosis of psychiatric disorder lifetime (*SCID-I*): yes, *n* (%)^e^		25 (24)	25 (42)	0 (0)	*p* < 0.001
Psychotropic medication lifetime: yes, *n* (%)^c,f^		23 (22)	20 (34)	3 (7)	*p* < 0.001
Psychotherapy lifetime: yes, *n* (%)^c^		25 (24)	19 (32)	6 (13)	*p* = 0.02
CTQ sum score, *M* ± *SD*		34.47 ± 10.19	39.93 ± 10.65	27.46 ± 2.13	*W* = 66.5, *p* < 0.001
Women with at least mild CM experiences in the following subscales, *n* (%)					
	Emotional abuse	23 (21)	23 (39)	0 (0)	*p* < 0.001
	Physical abuse	12 (11)	12 (20)	0 (0)	*p* < 0.001
	Sexual abuse	14 (13)	14 (24)	0 (0)	*p* < 0.001
	Emotional neglect	43 (41)	43 (73)	0 (0)	*p* < 0.001
	Physical neglect	14 (13)	14 (24)	0 (0)	*p* < 0.001

CM = childhood maltreatment; CTQ = Childhood Trauma Questionnaire^23,24^; *SCID-I* = Structured Clinical Interview^55^.

^a^Missing data for one woman of the CM group.

^b^Only chronical diseases or taken medication with more than one occurrence are displayed.

^c^Missing data for two women of the CM group.

^d^Self-reported lifetime psychiatric diagnoses: major depressive disorder (MDD; *N* = 4), anxiety disorders (*N* = 3), other diagnoses with one occurrence each (anorexia, adjustment disorder; comorbid: bulimia & PTSD, anxiety disorder & MDD).

^e^In *SCID-I* interview diagnosed lifetime psychiatric diagnoses according to *DSM5* criteria: MDD (*N* = 4), anxiety disorders (*N* = 11), alcohol use disorder (*N* = 2); comorbid diagnoses: MDD & anxiety disorder (*N* = 2), MDD & PTSD & anxiety disorder (*N* = 2), MDD & PTSD (*N* = 1), MDD & alcohol use disorder (*N* = 1), MDD & stimulant use disorder (*N* = 1), MDD & social phobia & obsessive-compulsive disorder (*N* = 1).

^f^Lifetime psychotropic medication with more than one occurrence: Hypericum perforatum (*N* = 7 in CM group), Valeriana (*N* = 3 in CM group), Lorazepam (*N* = 3 in CM group, *N* = 1 in control group).

^g^For normally distributed continuous data *t*-test is presented, if residuals were not normally distributed Mann-Whitney-*U*-test is displayed, and for categorical data Fisher’s exact test.

### Biomarker signature development: Univariate statistics

A combination of univariate (group comparisons & correlations) and multivariate methods (classification algorithms) was performed^26^. The univariate comparison of women with and without CM revealed 37 compounds as potential biomarker candidates with an original *p* value of ≤0.05. Eight of these metabolites met the False Discovery Rate (FDR)^27^ criterion of ≤0.10, thus survived multiple testing correction and should be seen as most important (see Table 2). Three compounds were increased and five compounds were decreased in the CM group compared to the control group. According to METLIN database, bilirubin IXa, the prostaglandin PGH_2_-EA, and the glycerolipid DG(18:0/20:3/0:0) match the accurate mass of the compounds that were found to be increased in the CM group, while ubiquinone 8 and the glycerophospholipids PA(O-18:0/12:0), PC(O-18:0/20:0), PI(20:0/20:4), and PI(22:2/20:5) match the accurate mass of the compounds that were observed to be decreased in the CM group.

**Table 2:** Results of univariate group comparisons.

Experiment	METLIN-match	MM (Da)	Rt (min)	Control group abundance score (log_2_)		CM group abundance score (log_2_)		Regulation in CM	Statistic^a^	FDR
				*N*	*M* (*SD*)	*N*	*M* (*SD*)			
Lipids, positive ionization mode	PC(O-18:0/20:0)	803.6784	8.65	42	20.97 (1.38)	44	18.33 (1.59)	↓	*t*(83.23) = 8.25, *p* < 0.001	<0.001
	Ubiquinone 8	726.5553	8.65	41	23.04 (1.11)	34	20.56 (1.76)	↓	*t*(53.7) = 7.12, *p* < 0.001	<0.001
	PI(22:2/20:5)	936.5721	3.94	30	18.06 (0.16)	37	17.88 (0.16)	↓	*t*(61.43) = 4.6, *p* < 0.001	<0.01
	DG(18:0/20:3/0:0)	646.5508	8.65	28	20.97 (0.25)	36	21.21 (0.16)	↑	*t*(43.9) = −4.42, *p* < 0.001	0.01
	PA(O-18:0/12:0)	606.4624	7.20	43	19.06 (0.37)	54	18.82 (0.32)	↓	*W* = 1604, *p* < 0.01	0.07
Lipids, negative ionization mode	PI(20:0/20:4)	914.5876	3.92	38	17.2 (0.46)	40	16.68 (0.4)	↓	*t*(72.94) = 5.33, *p* < 0.001	<0.001
Metabolites, positive ionization mode	PGH_2_-EA	393.2867	5.11	46	17.38 (0.23)	59	17.59 (0.41)	↑	*t*(93.31) = −3.32, *p* < 0.01	0.07
Metabolites, negative ionization mode	Bilirubin IXa	584.262	6.71	46	17.21 (0.92)	56	17.85 (0.96)	↑	*t*(97.75) = −3.45, *p* < 0.001	0.05

Abundance scores quantify the metabolite and lipid fractions. Only METLIN database-matched compounds with significant group comparisons after multiple testing correction with a False Discovery Rate (FDR) ≤0.10 are displayed; CM = childhood maltreatment; CTQ = Childhood Trauma Questionnaire^23,24^; MM = Monoisotopic mass in Da; Rt = Retention time in min.

^a^For normally distributed continuous data Welch’s *t*-test was calculated, if residuals were not normally distributed Mann-Whitney-*U*-test was computed.

In addition, the abundance of two accurate mass-matched compounds correlated significantly with the cumulative score of CM experiences (CTQ sum score, representing *maltreatment load*)^28^, even after multiple testing correction: phosphatidylcholine PC(O-18:0/20:0) (Kendall’s *τ* = −0.37, *p* < 0.001, FDR <0.001) and ubiquinone 8, also known as coenzyme Q_8_, (Kendall’s *τ* = −0.36, *p* < 0.001, FDR <0.01). However, all these accurate mass matches require structural validation for compound identification.

### Biomarker signature development: Multivariate statistics

In contrast to univariate approaches, multivariate classification algorithms have the advantages that they take the multivariate nature of the data into account, do not have to consider corrections for multiple comparisons, and can hence investigate patterns of correlated metabolites, which are related to CM. By simultaneously investigating a large set of metabolites, they can identify the best biomolecular panel differentiating between women with and without CM. We applied two multivariate classification algorithms: Partial Least Square Discriminant Analysis (PLS-DA), combining dimension reduction and classification approaches, as the state of the art classification method in metabolomics science, and Random Forests embedded in a Conditional Inference framework (RF-CI) as an alternative method with higher performance in variable selection (^29^; for a detailed description see Supplementary Methods). Both methods are particularly suited for the multivariate data structure and can handle highly inter-correlated predictors (as is the case for metabolites). Combining these two methods in a complementary approach was suggested in the literature^29,30^. The study cohort was randomly separated to a training set (3/4 of whole sample; *N*_train_ = 79 with 46 CM+ and 33 CM−) and a validation set (1/4 of whole sample; *N*_validation_ = 26 with 13 CM+ and 13 CM−), which did not differ significantly in demographic and clinical characteristics (all *p* values > 0.05). The following indicators for significance of a metabolite in differentiating between the absence and presence of a history of CM were calculated: *variable importance in projection* (VIP) scores in PLS-DA and *conditional variable importance* (*cvi*) values, representing the averaged mean decrease in accuracy in RF-CI.

**Figure 1:**
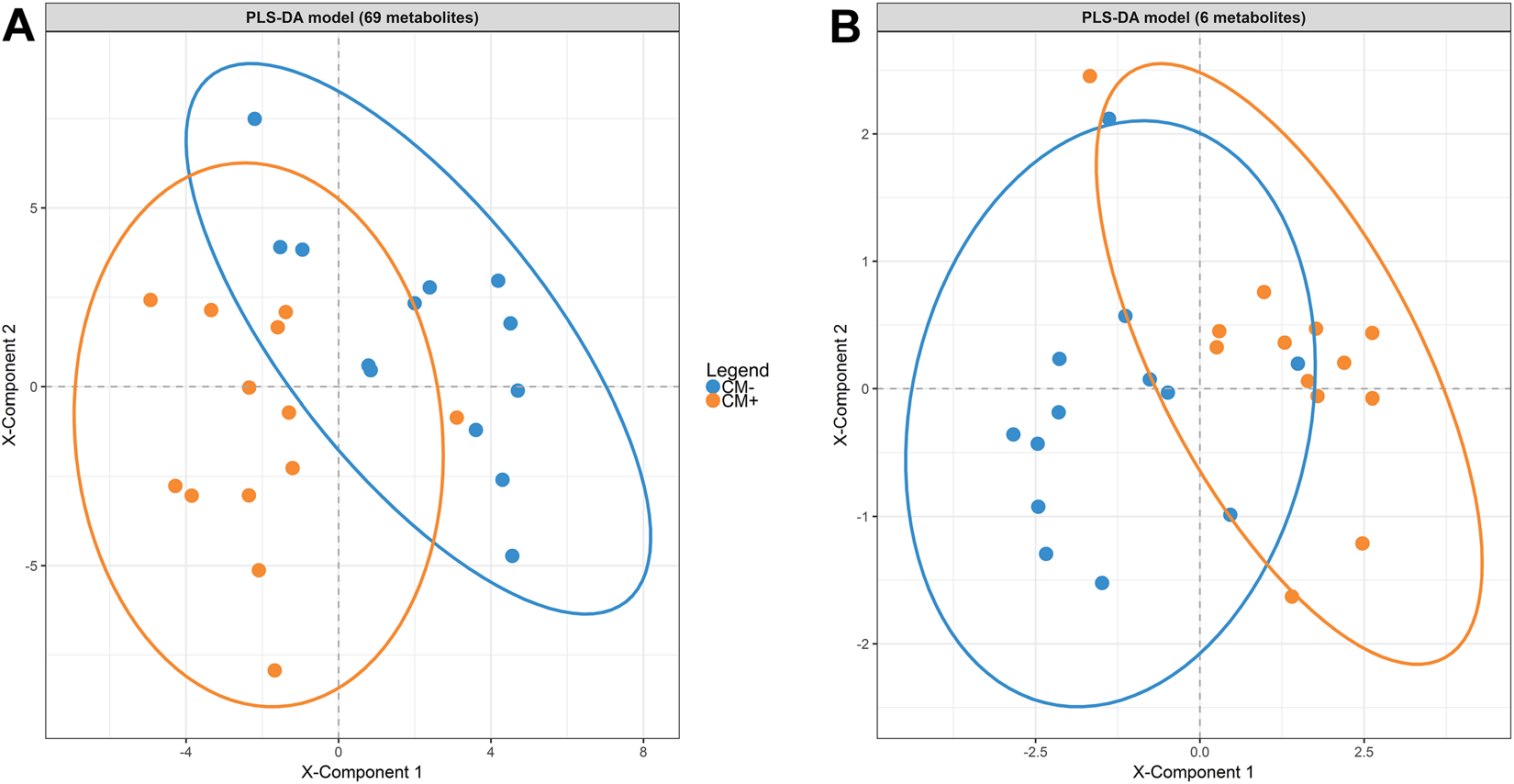
(**A** and **B**) Separation of women with (orange, *N* = 13) and without (blue, *N* = 13) childhood maltreatment (CM) experiences in the validation set based on the in cross-validation selected 3-component PLS-DA model with 69 metabolites (VIP threshold = 1.2, accuracy: 80.8%, sensitivity: 76.9%, specificity: 84.6%; **A**) or a more economical 3-component PLS-DA model with only six metabolites (VIP threshold = 2.5, accuracy: 88.5%, sensitivity: 92.3%, specificity: 84.6%; **B**).

A PLS-DA model with three components (see Supplementary Table S2) and 69 metabolites exceeding a VIP threshold of 1.2 was identified as the most suitable model as indicated by the best accuracy, the second best sensitivity, and the best specificity in a 10-fold, 1000 repeated cross-validation procedure in the training set (see Supplementary Figure S3). The application of the PLS-DA model with these 69 selected metabolites (see Table 3 for the first 30 metabolites with the highest VIP scores) predicted a history of CM in the validation set with an accuracy of 80.8%, a sensitivity of 76.9% and a specificity of 84.6%. The separation of the CM and the control group in the validation set based on three components comprising 69 metabolites is shown in Fig. 1A.

**Table 3:** Variable importance scores for the 30 top-ranked METLIN-matched compounds in PLS-DA and RF-CI predicting a history of CM.

Results of PLS-DA			Results of RF-CI		
Rank	METLIN-match [MM in Da; Rt in min]	VIP	Rank	METLIN-match [MM in Da; Rt in min]	*cvi* (%)
1	PC(O-18:0/20:0) [803.6784; 8.65] *	3.56	1	PC(O-18:0/20:0) [803.6784; 8.65] *	4.88
2	Ubiquinone 8 [726.5553; 8.65] *	3.12	2	Ubiquinone 8 [726.5553; 8.65] *	1.61
3	DG(18:0/20:3/0:0) [646.5508; 8.65] *	2.88	3	DG(18:0/20:3/0:0) [646.5508; 8.65] *	1.13
4	PA(O-18:0/12:0) * [606.4624; 7.20] *	2.79	4	PI(22:2/20:5) [936.5721; 3.94] *	0.86
5	PI(22:2/20:5) [936.5721; 3.94] *	2.63	5	PA(O-18:0/12:0) * [606.4624; 7.20] *	0.85
6	PI(20:0/20:4) [914.5876; 3.92] *	2.55	6	PI(20:0/20:4) [914.5876; 3.92] *	0.55
7	**PGH**_**2**_ -EA [393.2867; 5.11] *	2.29	7	**PGH**_**2**_ -EA [393.2867; 5.11] *	0.41
8	**TG(16:1/20:4/20:5) [898.7025; 10.26]**	2.18	8	**DG(22:4/22:4/0:0) [720.5697; 8.94]**	0.12
9	Bilirubin IXa [584.262; 6.71] *	2.15	9	**N-methyl arachidonoyl amine [317.2723; 5.13]**	0.08
10	**PE(18:0/0:0) [481.316; 7.64]**	2.13	10	**TG(16:1/20:4/20:5) [898.7025; 10.26]**	0.08
11	**LysoPE(0:0/22:0) [583.3842; 1.76]**	2.13	11	**PE(18:0/0:0) [481.316; 7.64]**	0.08
12	**N-methyl arachidonoyl amine [317.2723; 5.13]**	2.02	12	**4-Carboxy-4‘-sulfoazobenzene [306.031; 0.55]**	0.06
13	**C17 Sphinganine [287.282; 5.15]**	1.88	13	**LysoPE(0:0/22:0) [583.3842; 1.76]**	0.06
14	PS(22:0/0:0) [581.3689; 7.89]	1.70	14	**C17 Sphinganine [287.282; 5.15]**	0.05
15	Levulinic Acid, 3-Benzylidenyl- [204.0784; 7.40]	1.69	15	**(6RS)−22-oxo-23,24,25,26,27-pentanorvitamin D3 6,19 sulfur-dioxide adduct/(6RS)−22-oxo-23 [392.2022; 2.13]**	0.04
16	LysoPE(0:0/20:1) [507.3318; 7.89]	1.68	16	5β-Cholanic acid-3α, 12α-diol N-(2-sulphoethyl)-amide [499.2977; 3.55] *	0.03
17	PC(18:2/17:0) [771.5771; 5.30]	1.66	17	PA(O-16:0/19:0) [676.54; 6.11]	0.03
18	**DG(22:4/22:4/0:0) [720.5697; 8.94]**	1.65	18	**PI(18:2/0:0) [596.2955; 6.64]**	0.03
19	Glycerophospho-N-Arachidonoyl Ethanolamine [501.2846; 6.73]	1.62	19	Flupenthixol-O-glucuronide [625.2071; 4.78]	0.03
20	**(6RS)−22-oxo-23,24,25,26,27-pentanorvitamin D3 6,19 sulfur-dioxide adduct/(6RS)−22-oxo-23 [392.2022; 2.12**]	1.58	20	**Adenine [135.0544; 1.06]**	0.02
21	Methyl 8-[2-(2-formyl-vinyl)−3-hydroxy-5-oxo-cyclopentyl]-octanoate [310.1777; 5.48]	1.58	21	**Protoporphyrin IX [564.2746; 2.51]**	0.02
22	**4-Carboxy-4‘-sulfoazobenzene [306.031; 0.55]**	1.56	22	DG(18:1/18:3/0:0) [616.5045; 7.28]	0.02
23	**PI(18:2/0:0) [596.2955; 6.64]**	1.56	23	Lactosylceramide (d18:1/24:1) [971.724; 6.72]	0.02
24	1-Octadecanamine [269.3083; 7.38]	1.54	24	PC(P-16:0/14:0) [689.5347; 5.00]	0.01
25	L-Homocysteic acid [183.0202; 0.61]	1.54	25	Bilirubin IXa [584.262; 6.71] *	0.01
26	PC(17:2/16:0) [743.546; 4.63]	1.49	26	**PC(17:0/11:0) [677.4971; 4.08]**	0.01
27	**PC(17:0/11:0) [677.4971; 4.08]**	1.49	27	Octadecadienoic acid [280.2403; 1.67]	0.01
28	**Adenine [135.0544; 1.06]**	1.47	28	Glucosylceramide (d18:1/16:0) [699.5628; 4.92]	0.01
29	Deoxyguanosine [267.097; 1.06]	1.46	29	PE(16:1/P-18:1) [699.5199; 5.50]	0.01
30	**Protoporphyrin IX [564.2746; 2.50]**	1.45	30	Glucosylceramide (d18:1/22:0) [783.6573; 7.11]	0.01

The first 30 top-ranked metabolites for the Partial Least Squares-Discriminant Analysis (PLS-DA) and random forests embedded in a conditional inference framework (RF-CI) are presented, displaying the respective importance of the candidates in the prediction of class membership (CM+ or CM−). For PLS-DA, Variable Importance in Projection (VIP) is provided, whereas conditional variable importance (*cvi*), representing the averaged mean decrease in accuracy, is stated for RF-CI. Bold highlighted metabolites popped up in the top 30 of both PLS-DA and RF-CI. Metabolites marked with an asterisk became significant in the univariate group comparisons after multiple testing correction. Da = Dalton; MM = Monoisotopic mass; Rt = Retention time.

In order to enhance a potential clinical application, we further chose a more economical PLS-DA model with a higher VIP threshold. According to the performance criteria, a model with a VIP threshold of 2.5 consisting of only six metabolites – which also showed significant group comparisons after multiple testing correction in the univariate analyses – appeared as the best choice out of different economical models (see Supplementary Figure S3). This more economical model revealed an accuracy of 88.5%, a sensitivity of 92.3% and a specificity of 84.6% in the class membership prediction of the validation set (see also Fig. 1B), and consequently performed better in the validation set than the previously described PLS-DA model with 69 metabolites.

The calculation of 101 random forests, including all 398 candidates matched to endogenous biomolecules, predicted the class membership of all women in the validation set with an accuracy of 80.8%, a sensitivity of 92.3% and a specificity of 69.2%. The *cvi* values for the first 30 top-ranked metabolites are presented in Table 3.

According to the fitted models, the two metabolites database-matched by accurate mass as phosphatidylcholine PC(O-18:0/20:0) and ubiquinone 8 showed the highest variable importance scores in PLS-DA (VIP_(PE)_ = 3.56 and VIP_(U8)_ = 3.12) and in RF-CI (*cvi*_(PE)_ = 4.9% and *cvi*_(U8)_ = 1.6%) predicting past exposure to CM. To evaluate their single predictive accuracy, logistic regression analyses were applied. PC(O-18:0/20:0) predicted a history of CM in the validation set with an accuracy of 88.5%, a sensitivity of 92.3% and a specificity of 84.6%. The candidate molecule ubiquinone 8 showed accuracy, sensitivity and specificity values of 84.6% each.

According to variable importance scores VIP and *cvi*, biomolecule candidates were ranked. Comparing the top 30 metabolites of PLS-DA and RF-CI unveiled an overlap of 20 molecules (see Table 3) with the candidate metabolites with monoisotopic mass 803.6784 Da and retention time 8.65 min (METLIN-matched to the glycerophosphatidylcholine PC(O-18:0/20:0)) as well as with monoisotopic mass 726.5553 Da and retention time 8.65 min (METLIN-match: ubiquinone 8) displaying the greatest importance in the prediction of CM in both methods.

### Structural identification of candidate biomarkers by MS/MS

**Figure 2:**
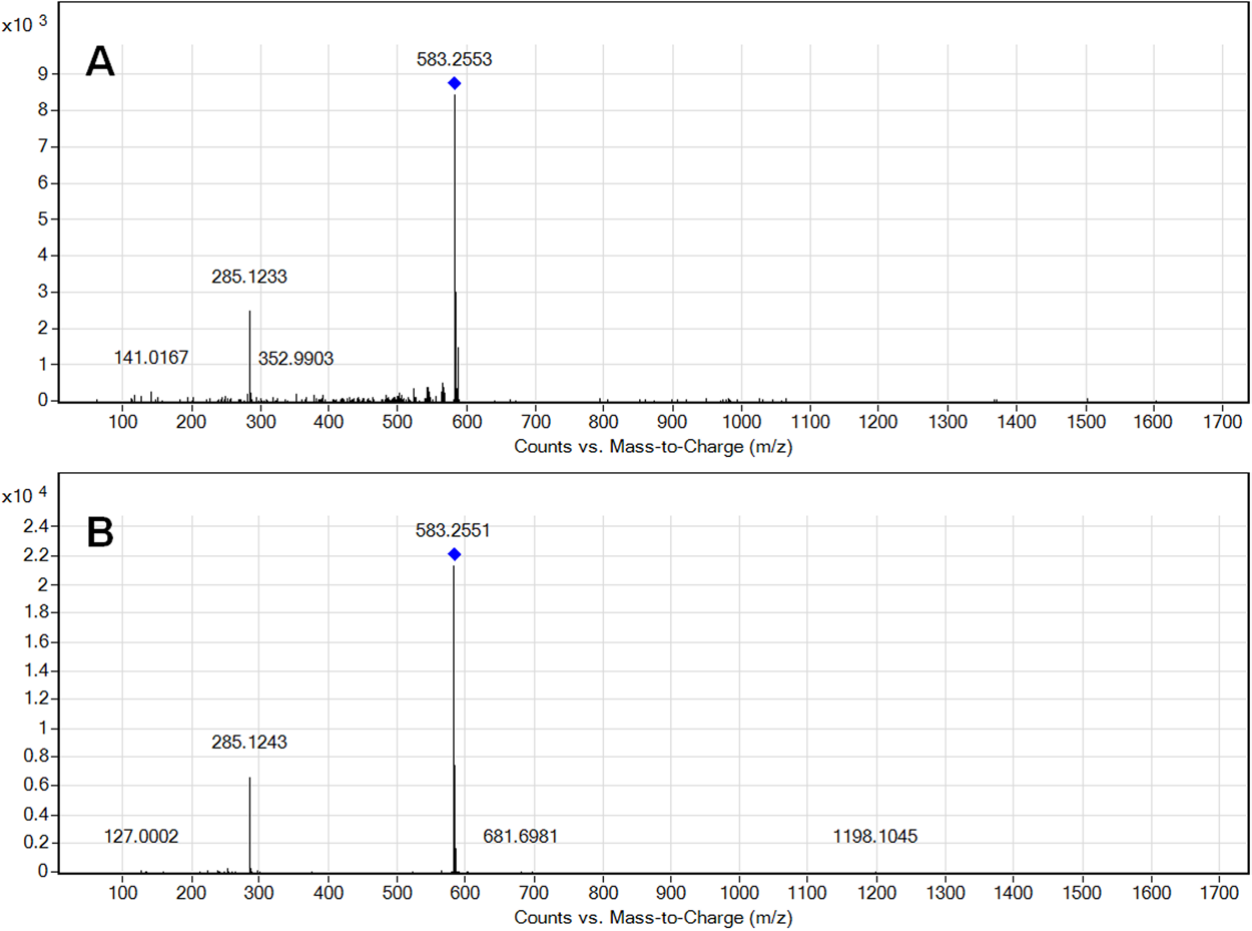
Comparison of bilirubin IXa MS/MS spectra between a pooled serum sample (**A**) and a commercial standard (**B**). Both fragmentation spectra show matches for residual precursor [M-H]^−^ (m/z 583) and a prominent fragment ion (m/z 285) with MS/MS data acquired at a fixed collision energy (CE) of 10 V and the mass spectrometer operated in negative ionization mode.

In the next step, we investigated the identity of the set of eight metabolite candidates shown to be significant in both the univariate and the multivariate approaches using tandem mass spectrometry (MS/MS): bilirubin IXa, PGH_2_-EA, ubiquinone 8_,_ PA(O-18:0/12:0), PC(O-18:0/20:0), PI(20:0/20:4), PI(22:2/20:5), and DG(18:0/20:3/0:0). MS-DIAL (version 2.54)^31^ was used to compare MS/MS fragmentation data obtained from LC-MS/MS runs at fixed collision energies (10, 20, 40 V) against in-built MS/MS reference library LipidBlast. In addition, selected metabolite fragmentation information was directly exported from MS-DIAL into MS-Finder (version 2.10)^32^ for further spectra comparison. MS-FINDER predicts molecular formulas based on MS information and retrieves isomer structures from metabolome databases (including HMDB, PubChem, ChEBi) to predict MS/MS spectra *in-silico*. Of the eight compounds targeted for MS/MS, three compounds (bilirubin IXa; ubiquinone 8; PC(O-18:0/20:0)) showed confident spectra similarities. In a last step, commercially available compounds were obtained for two metabolites (bilirubin IXa and ubiquinone 8). Comparison of the pure compound LC-MS/MS to our experimental LC-MS/MS confirmed the identification of bilirubin IXa (Fig. 2), but not ubiquinone 8. The identity of the candidate metabolite with monoisotopic mass 726.5553 Da and retention time 8.65 min, which was METLIN-matched to ubiquinone 8, and all of the other METLIN-matched candidates remain to be confirmed in future studies.

## Discussion

Combining the strengths of univariate and multivariate statistical approaches, we demonstrated for the first time the potential to elaborate serum-based biomolecular signature of CM experiences. In terms of a biomarker discovery approach, this study revealed novel antioxidant-, lipid- and endocannabinoid-associated pathways potentially related to the adverse health consequences of CM that were present even decades after CM exposure. Previously intensively investigated biomarkers for CM such as cortisol^33^ and kynurenine^34^ were measured, but had no discriminative power in our study (data not shown). Group differences in the abundance scores of the metabolites between women with and without CM experiences were found for eight biomolecule candidates (see Table 2). With regard to a potential dose-response-relationship, significant relationships between two biomolecule candidates (METLIN-matched to PC(O-18:0/20:0) and ubiquinone 8) and maltreatment load could be shown suggesting a cumulative effect of CM experiences on the human metabolic fingerprint.

In order to focus additionally on the high inter-correlations between metabolites and lipids and their orchestrated effects, a complementary approach combining PLS-DA and RF-CI was chosen to determine the best biomolecular panel with the highest power in discriminating women with and without CM experiences in a selected training set. Subsequently, the resultant PLS-DA and random forest models were evaluated in an independent validation set.

The RF-CI model including all 398 metabolite candidates leads to an overall good prediction of CM with a high percentage of women with CM experiences correctly categorized, but a rather low percentage of women without CM experiences correctly assigned. Following the cross-validation procedure in PLS-DA, the 3-component model with 69 metabolites showed the best performance criteria in predicting the group assignment of the training set. The evaluation of this model in the independent validation set resulted in the same predictive accuracy as the RF-CI model, but with higher specificity at the expense of poorer sensitivity. In the validation set, the most economical model with only the candidate metabolite with monoisotopic mass 803.6784 Da and retention time 8.65 min (METLIN-match: PC(O-18:0/20:0)) as a predictor produced the best performance criteria (accuracy, sensitivity, specificity) regarding the differentiation of women with and without CM experiences.

Collectively, univariate and multivariate results revealed a total of eight candidates that are individually significant after adjusting for multiple comparisons with FDR, and ranked top 30 in variable importance scores of the full PLS-DA and RF-CI models. One of these candidates increased in the CM group was structurally validated as bilirubin. In general, unconjugated bilirubin, as part of the heme metabolism, is a potent antioxidant with immunomodulatory properties^35^. Further, previous studies showed that bilirubin inhibits the synthesis of reactive oxygen species in vascular cells and thereby potentially prevents CM-associated diseases such as coronary heart disease^36^. Higher levels of serum bilirubin were also found for post-stroke depression and were interpreted to reflect the intensity of initial oxidative stress^37^. Metabolite profiling in PTSD suggested bilirubin as potential biomarker differentiating between PTSD cases and controls^20^. In line with these results, the current findings support an involvement of bilirubin after CM experiences by potentially counteracting the adverse effects of oxidative stress.

According to accurate mass matching of METLIN database, four of the eight significantly altered metabolites belong to the glycerophospholipid class of lipids, and were all decreased in CM. Lipids are important structural components of cellular membranes, but also participate in cellular signaling and energy storage^38^. In line with our results, recent studies found altered lipid levels for stress-related psychiatric disorders, such as MDD^39,40^ and PTSD^20^. The molecular identities of these candidate lipids remain to be verified.

Although structural confirmation is pending, this study also revealed a significant elevation of a compound matched to PGH_2_-EA by accurate mass. PGH_2_-EA is part of the endocannabinoid inactivating pathway, and can result from the oxidation of endocannabinoid anandamide (AEA) by *cyclooxygenase-2*^41,42^. AEA has been shown to exhibit neuroprotective, anti-inflammatory^43^, but also neurotoxic effects that in turn lead to potential proinflammatory responses in metabolism^44^.

While further investigation is required to confirm PGH_2_-EA increase in individuals with CM, and the impact of increased PGH_2_-EA on endocannabinoid turnover, changes in this pathway have broad impact on health. In general, the interplay between endocannabinoid biosynthesis and inactivation determines the endocannabinoid system activity^42^, which in turn is controlling appetite, food intake and energy balance through glucose metabolism^45^. Further, the endocannabinoid system has been reported as a key modulator of pain regulation^46^, and to play a role in the stress response regulation, fear and anxiety coping by modulating synaptic transmission processes (for a review see ref.^47^). Endocannabinoid signaling was suggested as a potential contributor to PTSD etiology in a recent metabolite profiling study^20^. Additionally, hair concentrations of endocannabinoids were associated with PTSD symptom severity^48^ hinting towards endocannabinoid-driven pathways in the aftermath of traumatic life events. With respect to chronic health effects in the aftermath of CM, individuals with a history of CM are at increased risk of experiencing chronic pain in adulthood^49^ and having a decreased pain tolerance^50^. We recently reported an association of CM with increased respiratory activity of mitochondria in intact immune cells and a pro-inflammatory phenotype in adulthood^51^. Future studies should determine whether these CM physiological effects are related to PGH_2_-EA increase and its impact on the endocannabinoid system.

A history of CM is associated with a higher risk for developing a psychiatric disorder^2^. In line with this, a high percentage of our CM group was diagnosed with a lifetime psychiatric disorder applying the *SCID-I* interview (42%, *N* = 25; see Table 1). As CM and a lifetime psychiatric disorder are so highly correlated, the exclusion of these 25 CM+ women would have resulted in an artificial cohort and hence, we refrained from excluding these women in the first place. However, to assure that our results are not merely driven by the higher proportion of lifetime psychiatric diagnoses in the CM+ group, we repeated our analyses excluding these 25 women with a *SCID-I*-diagnosed lifetime psychiatric disorder from the CM group (new group sizes: *N* = 34 CM+, *N* = 46 CM−) and obtained similar results (see Supplementary Information S7). Thus, we can conclude that our results are not simply driven by the present lifetime psychiatric diagnoses, but are associated with the exposure to CM.

### Strengths and limitations

The human metabolome is affected by both physiological and environmental factors, such as age^16^, parity (for pregnancy-related changes see e.g.^52^) and smoking behavior^21^, hence, discovery studies need to control for these factors. One strength of the study is the investigation of a rather homogenous group of Caucasian, adult, non-smoking, 3-months postpartum women from the same geographical region. Although this design eliminates potential confounding factors, it also limits the generalizability of results. Therefore, the validity of the discovered biomolecular signature needs to be confirmed in men and in women who are not pregnant or not in the postpartum period. Further, to replicate and complement our findings in serum metabolome, an investigation of the metabolite fingerprint of CM in other biofluids (e.g. saliva, urine) and in a longitudinal approach seems to be reasonable. In addition, the molecular identities of all candidate metabolites (apart from bilirubin IXa) remain to be confirmed.

The retrospective report of CM might go along with a significant under-reporting and measurement bias^53^. Yet, the results of this study argue for a nevertheless observable effect of retrospectively reported CM experiences on the metabolome. Lastly, even though the CM group and the control group did not differ in the intake of medication (see Table 1) and all metabolites and lipids of exogenous origin were excluded from the analyses, the potential influences of the taken medication on the metabolome cannot be completely excluded.

## Conclusion

For the first time, we were able to show the feasibility of a biomarker discovery approach in the context of CM in postpartum women. Metabolite fingerprinting in postpartum women with and without a history of CM revealed a biomarker signature that could explain long-term effects of CM on health and disease by influencing oxidative stress and inflammation. The biomarker signature achieved a clear differentiation between women with and without CM experiences with high accuracy rates (~80–90%). Thus, our study empirically strengthens the perspective that CM experiences initiate biomolecular pathways that might increase the biochemical vulnerability for mental health conditions later in life. Strikingly, similar pathways were observed in chronic stress and trauma-related conditions (including MDD and PTSD). Further replication studies are needed in independent cohorts. The clinical value of the biomolecular signature and the related physiological mechanisms can then be investigated. Prospectively, this deeper understanding of underlying biomolecular processes could help to identify individuals at risk and to allocate preventive interventions.

## Materials and Methods

### Study participants and study design

The data was collected within the “My Childhood – Your Childhood” study, investigating risk and resilience factors in the transgenerational transmission of CM. The study was approved by the Ethics Committee of Ulm University and was performed in accordance with relevant guidelines and regulations. Immediately after parturition (on average 2.7 days [*SD* = 4.8] after birth), 548 women were recruited at the maternity ward of the Ulm University Hospital and provided written informed consent for study participation. Out of the initial cohort, 533 women participated in a screening for CM using the German version of the *Childhood Trauma Questionnaire* (CTQ)^23,24^. The cut-off criteria of the CTQ^54^ were used to divide the women in two groups: a control group including women without CM experiences (CM−) and a group of women reporting at least mild to severe maltreatment experiences during childhood (CM+). Three months postpartum, 285 women participated in a follow-up that consisted of a psychodiagnostic interview and a sampling of peripheral blood. The psychodiagnostic interview included the comprehensive assessment of socio-demographic, medical and clinical variables as well as the German research version of the *Structured Clinical Interview* (*SCID-I*)^55^ for the diagnosis of (current and lifetime) major axis I disorders of the *Diagnostic and Statistical Manual of Mental Disorders, Fourth Edition* (4th ed., text rev.; *DSM-IV-TR*)^56^. With the publication of the *DSM-5*^57^, some diagnostic criteria changed. As the to *DSM-5* changes updated version of the *SCID-I* has not yet been published when the study started, expert clinicians formulated additional questions and instructions to allow diagnoses of the mental disorders tested according to *DSM-5* (e.g. bereavement was no exclusion criteria within the diagnosis of MDD in *DSM-5*). To further assess the lifetime exposure to traumatic events, the German version of the *Posttraumatic Stress Diagnostic Scale* (PDS)^58^ event list was used.

The sampling of peripheral blood for the preparation of serum was performed in 247 women. Due to the usage of the blood samples in other biological analyses, only 222 of these blood samples were available for the metabolomics analyses. Out of these 222 available samples, women were excluded due to missing psychological data (*N* = 30), autoimmune disease (*N* = 9), current smoking (*N* = 9), non-Caucasian ethnicity (*N* = 1), obesity (BMI >30 kg/m²; *N* = 18), acute intake of psychotropic medication (*N* = 1), and acute illness (*N* = 18). Women in the control group were also excluded if they had had a lifetime history of a psychiatric disorder (*N* = 23) or severe distress within the last three months (e.g. death of a close person; *N* = 8). Thus, the final study cohort consisted of 46 women in the control group (CM−) and 59 women in the CM+ group.

The women reported their CM experiences retrospectively, which potentially led to significant under-reporting and measurement bias^53^. To consider these potential effects, the Minimization-Denial subscale of the CTQ was analyzed as suggested in the literature^59^ and identified eleven women, whose answers point towards a positive response bias. Nevertheless, all eleven women reported CM experiences, so they could not be falsely categorized as controls.

Detailed description of blood sampling, metabolite extraction, mass spectrometry, and statistical methods is available in Supplementary Methods. Data is available upon request from the corresponding authors.

## Electronic supplementary material

Supplementary Information
