# Supplementary material for: Serum profile changes in postpartum women with a history of childhood maltreatment: a combined metabolite and lipid fingerprinting study
Source: Sci Rep. 2018 Feb 22;8:3468. doi: 10.1038/s41598-018-21763-6 (PMC5823924; doi:10.1038/s41598-018-21763-6)
Supplement: Supplementary file 1 — Supplementary Information [file 41598_2018_21763_MOESM1_ESM.pdf]

Supplementary Information

Serum profile changes in postpartum women with a history of childhood maltreatment: a combined metabolite and lipid fingerprinting study

Alexandra M Koenig[a,*], Alexander Karabatsiakis[a,*], Thomas Stoll[b], Sarah Wilker[a], Thomas Hennessy[b,c], Michelle M Hill[b], Iris-Tatjana Kolassa[a]

[a] Clinical & Biological Psychology, Institute of Psychology and Education, Ulm University, Albert-Einstein-Allee 47, 89081 Ulm, Germany

[b] The University of Queensland Diamantina Institute, The University of Queensland, Translational Research Institute, 37 Kent Street, Woolloongabba QLD 4102, Australia

[c] Agilent Technologies, 679 Springvale Road, Mulgrave, VIC 3170, Australia

[*] These authors contributed equally to this paper.

Correspondence: Alexandra M Koenig and Alexander Karabatsiakis, Clinical & Biological Psychology, Institute of Psychology and Education, Ulm University, Albert-Einstein-Allee 47, 89081 Ulm, Germany. Telephone: 0049 731 5026597. Mail: alexandra.koenig@uni-ulm.de, alexander.karabatsiakis@uni-ulm.de

Supplementary Methods

Blood sampling, processing and metabolite extraction

Prior to the psychological interview, venous blood was drawn into 7.5 ml pre-chilled S-monovettes (Sarstedt, Nümbrecht, Germany) between 11 am and 2 pm. To avoid additional psychological strain, we did not ask participants to fast overnight. Directly after blood sampling, whole blood was centrifuged for 10 min at 3000 g and 4°C. Serum portions of 250 µl were aliquoted into pre-chilled tubes and immediately stored at -80°C.

After finalizing the sample collection, serum samples were thawed in randomized batches of 24 samples on ice for the extraction of metabolites and lipids. An aliquot of 200 µl of each sample was transferred into a new reaction tube and mixed with 600 µl of ice-cold chloroform/methanol (1:2, v/v, both HiPerSolv Chromanorn, VWR International, Belgium, High Pressure Liquid Chromatography (HPLC)-certified). Reaction tubes were vortexed three times for 1 min with 5 min incubation time at 4°C in between (monophasic compound extraction). Then, 300 µl of ice-cold chloroform was added followed by mixing for 30 sec and a 10 min incubation on ice. Next, 200 µl of ice-cold ddH_2O was added to achieve phase separation followed by mixing for 1 min and centrifuging for 5 min at 14,000 rpm. From the resulting biphasic solution, 200 µl of the lower chloroform phase (lipids) and 200 µl of the upper methanol phase (metabolites) were transferred into separate reaction tubes and stored at -80°C. Subsequently, samples were dried in a CentriVap concentrator (Labconco, USA) and stored at -80°C prior to shipment to the University of Queensland Diamantina Institute (Brisbane, Australia) for analysis.

UHPLC-QTOF analysis

For LC/MS analysis, dry metabolite and lipid samples were re-suspended in 40 µl milliQ water on ice and 200 µl of methanol/toluene (9:1, v/v) at 4°C, respectively, followed by centrifugation for 15 min at 15,000 x *g* and 4°C. A supernatant aliquot was transferred into a vial glass insert and 2 µL injected onto the respective column. All lipid sample handling procedures were performed in a cold room (4°C) due to the relatively high volatility of solvents used. The metabolomics LC/MS platform consisted of a 1290 Infinity II UHPLC coupled to a 6550 iFunnel Q-TOF mass spectrometer via Dual AJS ESI source (Agilent, Santa Clara, USA). Separation of metabolites and lipids was performed on a Poroshell 120 EC-C18 (2.7 µm, 120 Å, 2.1 x 100 mm, pn 695775-902, Agilent) and a Zorbax Eclipse Plus C18 RRHD (1.8 µm, 95 Å, 2.1x50mm, pn 959757-902, Agilent) column, respectively. Each column was connected to a 2.1 x 5 mm guard column with respective resin. The autosampler temperature was set to 4°C. A needle wash solution of 50% and 100% 2-propanol was used for metabolite and lipid analysis, respectively, with a flush port time set to 15 s.

Chromatographic separation of metabolites comprised the following eluents and gradients: In positive ionization mode, eluent A was milliQ water containing 0.1% formic acid and eluent B was acetonitrile containing 0.1% formic acid. In negative mode, eluent A was 5 mM ammonium acetate (pH neutral) in milliQ water and eluent B was 5 mM ammonium acetate (pH neutral) in acetonitrile/milliQ water (90:10, v/v). Total method runtime was 15 min with the following gradient for both modes: 0 min (5% eluent B) - 9.5 min (100% B) – 10.5 min (100%B) – 10.6 min (5% B) - 15 min (5% B). Lipid separation was performed using the following conditions: In positive ionization mode, eluent A was acetonitrile/milliQ water (60:40, v/v) and eluent B was 2-propanol/acetonitrile (90:10,

v/v) with both eluents containing 10 mM ammonium formate and 0.1 % formic acid. In negative mode, ammonium formate and formic acid was replaced with 10 mM ammonium acetate (pH neutral) in both eluents. The following gradient was employed for both modes: 0 min (15% eluent B) - 2 min (30% B) – 2.5 min (48%B) – 11 min (82% B) – 11.5 min (99% B) - 12 min (99% B) – 12.1 min (15% B) – 15 min (15% B). A flow rate of 0.5 mL/min and column temperature of 60°C was applied in all LC methods.

The LC/MS platform was controlled using MassHunter data acquisition software version B.06.01 (Agilent) and the mass spectrometer was tuned and/or check tuned regularly in positive and negative ionization mode using the Agilent tuning mix (pn G1969-85000) in the following instrument state: Mass range 'Low (1700 m/z)', instrument mode 'Extended Dynamic Range (2 GHz)' and slicer mode 'High Sensitivity'. The tune report was checked for TOF mass calibration error and resolution, profile and intensity of tune ions, quadrupole isolation efficiency as well as contamination in the low mass range. During data acquisition a solution (pn G1969-85001) with reference ions for positive (m/z 121.050873, 922.009798) and negative ionization mode (m/z 68.995758, 112.985587, 1033.988109) was continuously infused through the second sprayer of the Dual AJS source for automatic TOF mass re-calibration. All data was stored as centroid.

Untargeted discovery experiments were performed by acquiring full scan MS spectra (m/z 50-1700) at a scan rate of 2 and 2.5 spectra/sec for metabolites (equals 4059 transients/spectrum) and lipids (equals 3224 transients/spectrum), respectively, with the following settings: Gas temperature 250°C, gas flow 15 L/min, sheath gas temperature and flow at 400°C and 12 L/min, respectively, nebulizer 30 psi, fragmentor 365, capillary voltage at 4000 V for positive and 3500 V for negative mode, nozzle voltage and collision energy were zero.

A quality control (QC) sample was generated by pooling 4 µl of each sample. This sample pool served as a technical QC (TQC) to test for instrument variation (retention time and signal stability). Before a sample sequence, 6 replicate injections of 2µL TQC were performed to condition the column. Then, TQCs were injected in duplicate at the beginning and end of each sequence and after every set of 10 samples. All samples were measured in batches with randomized order and no blank injections were performed between samples. Additionally, dilutions of the TQCs (1:1, 1:2, 1:5, 1:10) were measured to check if the m/z of a compound of interest diminishes when diluted (see Supplementary Figures S5 and S6).

For compound identification after data processing and statistical analysis, the 'Auto MS/MS' acquisition mode with a 'Preferred/Exclude' table was employed, i.e. only compounds of interest compiled in an inclusion list containing precursor mass and retention time information were targeted for MS/MS. Inclusion lists were comprised of up to 130 compounds. The following data acquisition settings were used: MS and MS/MS mass range was 50-1700. MS and MS/MS acquisition rate was 5 spectra/sec (equals 1552 transients/spectrum) and 3 spectra/sec (2583 transients/spectrum), respectively. A maximum of 5 precursors were targeted per cycle which resulted in a cycle time of 2 s. Quadrupole isolation width was ~4 amu (medium). Each sample was run in triplicate with a fixed collision energy (CE) of 10, 20 and 40 V each. Additional precursor settings: Threshold of 10,000 counts, exclusion after 1 spectra for 0.5 min, delta mass tolerance of 15 ppm, delta retention time tolerance of 0.25 min and charge state of 1. Reference ions were excluded from fragmentation with a delta mass tolerance of 5 ppm. Funnel voltage values were kept at 50, 200 and 100 V for Exit DC, RF HP and RF LP, respectively. For MS/MS data acquisition of commercial standards aforementioned 'inclusion list method' was slightly modified. Active precursor exclusion was disabled and changes to the

'Preferred/Exclude' table were as follows: delta mass tolerance of 20 ppm and delta retention time tolerance of 2 min.

Raw data pre-processing and quality control

The created data files of the 105 samples were loaded into *MassHunter Profinder* (version B.06.00, Agilent Technologies) for compound feature extraction (CFE). Using the algorithm of the provided batch recursive feature extraction wizard, a retention time window of 0 % + 0.15 min and a mass window of 20 ppm + 2.00 mDa were selected as binning and alignment tolerances. Features (metabolites/lipids) must satisfy these filter conditions in at least 50 % of all 105 samples, presenting at least 5000 counts for each compound. This recursive analysis was performed to reduce the number of false missing values for individual compounds. Recursed data were exported as CEF-files and loaded into *Mass Profiler Professional Software* (MPP; version 14.5, Agilent Technologies). In positive ionization mode, a total of 1062 metabolite compounds and 1077 lipid compounds was found, whereas with 391 metabolite and 331 lipid spectra in the negative ionization mode the total amount of compounds was much lower. Via the software *ID Browser* (version B.07.00, Agilent Technologies), spectra for metabolites and lipids were matched to the information available from the METLIN Metabolomics Database (The Scripps Research Institute, USA). METLIN provides a repository of metabolite and lipid information together with mass spec data for more than sixty-four thousand bioactive molecules. Identified compounds were separated manually from the unidentified, leading to lists of 211 metabolite and 167 lipid candidates for positive ionization mode. In negative ionization mode, 167 metabolite and 129 lipid candidates were determined. In a manual filtering process, metabolites and lipids of exogenous origin (e.g. drugs, cosmetics, plant nutrients and products, and environmental compounds) were removed from these

lists in all conscience. The remaining entities must be present in at least 50% of the samples within both groups (CM+ and control group), resulting in final lists of 104 metabolite and 131 lipid candidates (positive ionization mode) as well as of 102 metabolite and 117 lipid candidates (negative ionization mode). In a final step, these four lists were merged for statistical analyses. Before merging was possible, duplicates (*N*= 47) and quadruplicates (*N*=3) of candidate compounds were identified. One of each duplicate or quadruplicate was selected by applying the following criteria: 1) its presence in the greatest sample size and 2) the largest abundance. The final data set, which consisted of 398 metabolite and lipid candidates, was loaded into R for statistical analyses.

Identification of biomarker candidates by MS/MS fragmentation

To confirm biomarker candidates, a MS/MS inclusion list (precursor mass, retention time) was generated using MPP (part of GeneSpring software) through non-adjusted univariate statistics (Student's *t*-tests with $p<.05$). LC-MS/MS analyses were performed on the pooled sample used as QC with a fixed CE of 10V, 20V and 40V per run. The resulting fragmentation data was analyzed using MS-DIAL (version 2.54) and MS-FINDER (version 2.10). The results of this work were independently conducted and did not influence the number of biomarker molecules to be tested in the statistics.

Further confirmation was performed for two candidates by comparing their fragmentation data to commercially available standards, namely bilirubin IXa (metabolite fraction, negative ionization mode; Sigma pn 14370) and ubiquinone 8 (lipid fraction, positive ionization mode; Avanti Polar Lipids pn 900151). Bilirubin and ubiquinone 8 standards were dissolved in chloroform/methanol (1:1, v/v) and chloroform, respectively. Reference compounds were injected at different concentrations and MS data

was acquired to assess sample loading, retention time, precursor mass, and signal intensity. Subsequently, standards were injected in triplicate to acquire MS/MS data at a fixed CE of 10, 20 and 40 V each and fragmentation spectra were compared against spectra from discovery experiments. Chromatographic systems used for bilirubin and ubiquinone 8 analysis were as described above for metabolites in negative mode and lipids in positive ionization mode, respectively.

Statistical analyses

Statistical analysis of raw data for the database-matched metabolites and lipids of predicted endogenous origin was conducted using R version 3.3.0 (1). Continuous variables within the demographic and clinical characteristics of women with and without CM were compared by *t*-tests, if residuals were normally distributed, and by Mann-Whitney *U* tests for not normally distributed residuals. Fisher's exact test was chosen to compare categorical data. According to the established practice in metabolomics studies (2), a combination of univariate and multivariate methods was applied to study metabolic differences in abundance scores between women with and without CM experiences.

Concerning univariate analyses, once again *t*-tests were conducted for normally distributed residuals, and Mann-Whitney *U* tests were chosen, if residuals were not normally distributed. In addition, correlations between the CTQ sum score as an operationalization of the maltreatment load (cumulative score of CM experiences; 3) and the abundance scores of each compound were calculated using Kendall's τ. For all univariate analyses, missing values were omitted and the critical p value for statistical significance was set to .05. To counteract the risk of false positives, multiple testing correction is recommended as a necessary step in the metabolomics data analysis (2).

However, traditional methods like the Bonferroni adjustment have been seen as far too conservative for biomarker discovery approaches and the application of the False Discovery Rate (FDR; 4) is considered more appropriate (5-6). Thus, original *p* values (*N*=398 multiple comparisons) were adjusted by FDR. In line with these considerations, all significant metabolites with an adjusted *p* value (FDR) <.10 are displayed as potential biomarker candidates (cf. 7).

In contrast to univariate approaches, multivariate classification algorithms provide the advantage to take the multivariate nature of the data into account, and can hence investigate patterns of correlated metabolites, which are related to CM. By simultaneously investigating a large set of metabolites, they can identify the best biomolecular panel differentiating between women with and without CM and do not have to consider corrections for multiple comparison. We applied two multivariate classification algorithms: the Partial Least Square Discriminant Analysis (PLS-DA), combining dimension reduction and classification approaches, as the state of the art classification method in metabolomics science, and random forests embedded in a conditional inference framework (RF-CI) as an alternative method with higher performance in variable selection (8). Both methods illustrate the multivariate data structure and could appropriately handle highly inter-correlated predictors (as is the case for metabolites). Combining these two methods in a complementary approach was suggested in the literature (8-9). In a data pre-processing step, abundance scores were normalized and missing values were imputed using the non-linear iterative partial least square (NIPALS) algorithm (10-12) implemented in the R package "mixOmics 6.0.1" (13) since cross-validation cannot deal with missing values. Finally, the study cohort was randomly separated in a training set (3/4 of whole sample; N_{train}=79 with 46 CM+ and 33 CM-) and a validation set (1/4 of whole sample; $N_{validation}$=26 with 13 CM+ and 13 CM-), which did

not differ significantly in demographic and clinical characteristics (all p values >.05). For the evaluation of the prediction in the independent validation set by the fitted PLS-DA and RF-CI models, accuracy, sensitivity, and specificity were calculated. Sensitivity, also called *true positive rate*, represents the proportion of all women with CM experiences that were correctly assigned to the CM group, whereas specificity, the *true negative rate*, is defined as the percentage of all women without CM experiences correctly categorized as controls. Consequently, the percentage of accuracy comprises all correctly categorized cases and controls within the whole sample.

A comprehensive description of PLS-DA with regard to metabolite fingerprinting can be found elsewhere (7). Briefly, PLS-DA models extracting one to five key components were fitted for the training set and a 10-fold cross-validation was repeated 1000 times for each model to select the best, parsimonious model according to the highest level of predictive accuracy. Due to additional application of the one-standard error (*SD*) rule (14), we chose the model with three components with an accuracy less than one SD away from the 4-component model encompassing the highest predictive accuracy (see Supplementary Table S2). For the selected 3-component model, *variable importance in projection* (VIP) scores were calculated to identify metabolites with the most significant contribution in discriminating CM+ and CM-. As reported in the literature, metabolites with a VIP score greater than 1.0 should be selected for further analyses, but higher VIP thresholds are also discussed (15). To find the most appropriate VIP threshold according to our data, models with different VIP thresholds ranging from 1.0 to 2.75 were compared (see Supplementary Figure S3). The 3-component model with metabolites exceeding the most appropriate VIP threshold of 1.2 was employed to predict the group assignment of the validation set.

In order to review and consolidate the results of the PLS-DA, we used random forests embedded in a conditional inference framework, controlling for inter-correlation between the metabolites as predictor variables (RF-CI) (R package “party”, version 1.0-25; 16-18). RF-CI represents another common multivariate classification algorithm (for a more detailed description of the method see 19). The procedures to fit RF-CI models were similar to the ones described by Conrad and colleagues. As suggested in the literature (18), different values of the number of previously selected splitting variables – called mtry – were compared to determine the best model fit. Compared mtry values ranged between the square root of the amount of metabolites ($\sqrt{398}$=20), which is the default for random forest classification and one third of the amount of metabolites (398*1/3=133). Simulations were repeated 101 times with different random number generator seeds. Random forests with 500 trees each were built using the training set. Best results were obtained for mtry=120, providing an optimal interplay between mean predictive accuracy, small SD, and a parsimonious nature in comparison to other mtry values tested (see Supplementary Figure S4) and this value was consequently used in further calculations. Similarly to PLS-DA, an ordered rank list of the conditional variable importance for each metabolite was computed (*cvi*; 18), displaying the respective importance of the metabolites in the prediction of class membership (CM+ or CM-). Finally, random forests were used to predict the class membership of the validation set. Sensitivity, specificity, and accuracy for these predictions were calculated.

In a final step, logistic regression analyses were applied for the two metabolites with the highest VIP scores in the PLS-DA model and the highest *cvi* according to the RF-CI to evaluate their single predictive accuracy.

Supplementary Table S2:

Comparison of PLS-DA models extracting 1 to 5 key components according to prediction performance criteria.

Number of key components	Sensitivity (%) M (SD)	Specificity (%) M (SD)	Accuracy (%) M (SD)
1	67.22 (4.54)	47.97 (6.84)	59.18 (4.09)
2	72.58 (3.86)	54.28 (4.46)	64.94 (2.97)
3	75.12 (4.00)	63.57 (5.33)	70.29 (3.34)
4	75.89 (3.84)	68.09 (4.95)	72.63 (3.23)
5	75.45 (3.81)	65.95 (4.51)	71.48 (3.01)

Calculations are based on 1000 repeats of 10-fold cross validation in the training set. All METLIN-matched candidates (*N*=398) are included. Due to the application of the one-standard error (SD) rule (14), the 3-component model with an accuracy less than one SD away from the 4-component model, representing the model with the highest predictive accuracy, was chosen.

Supplementary Figure S3:

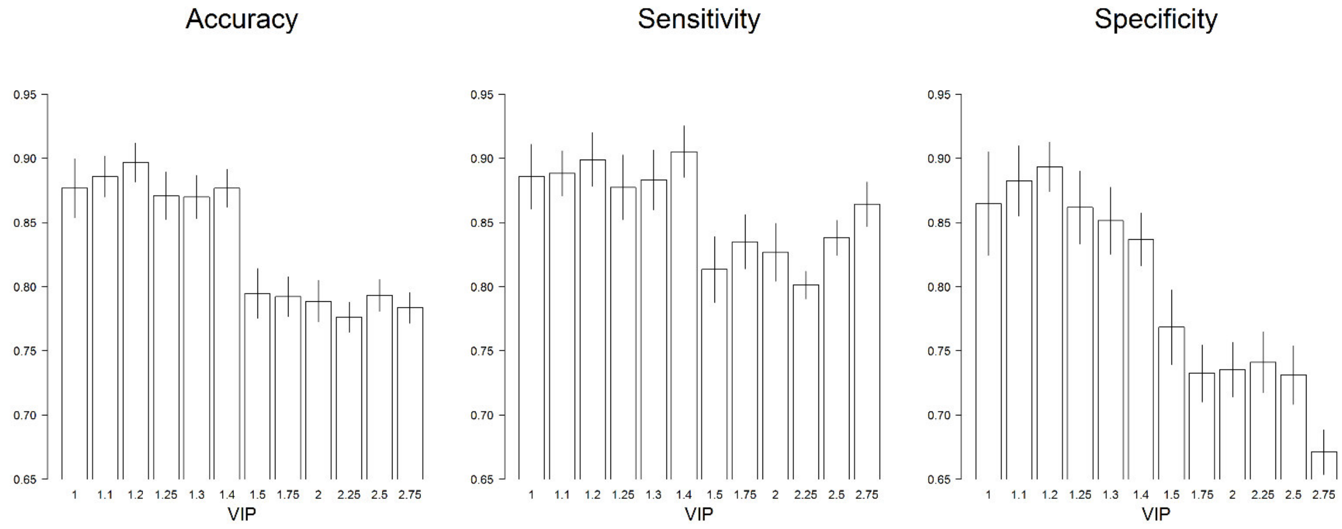

Prediction performance criteria for PLS-DA models applying VIP thresholds ranging from 1.0 to 2.75

Calculations are based on 1000 repeats of 10-fold cross validation in the training set. Means and standard deviations of the performance criteria are displayed for each model. The model with a VIP of 1.2 reached the highest predictive accuracy and specificity as well as a good sensitivity compared to the other models. Accuracy = percentage of correctly categorized cases and controls. Sensitivity = true positive rate. Specificity = true negative rate. VIP = Variable Importance in Projection threshold. PLS-DA = Partial Least Squares-Discriminant Analysis.

Supplementary Figure S4:

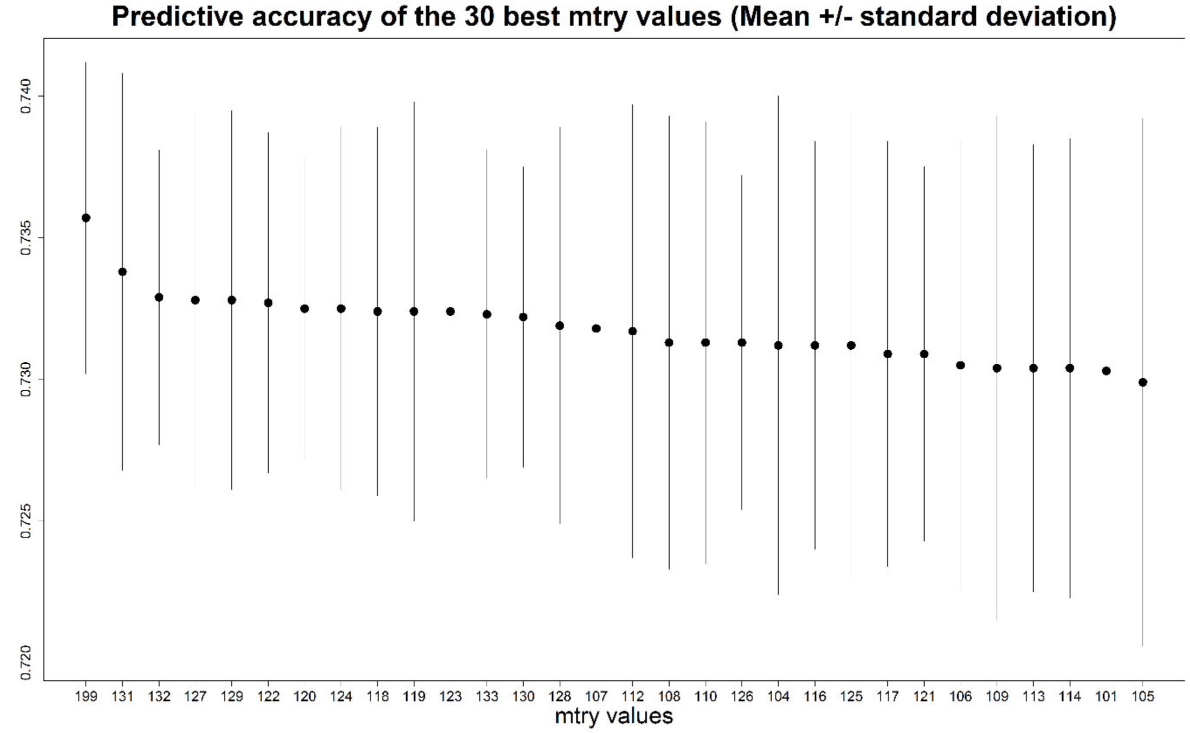

Calculations are based on 1000 repeats of 10-fold cross validation in the training set. Mtry represents the number of previously selected splitting variables in the construction of random forests. To determine the best model fit, different mtry values ranging from the square root of predictor variables ($\sqrt{398}$=20) to one third of the predictor variables (398*1/3=133) were compared. The 30 mtry values with the highest predictive accuracy are presented in the figure. Best results were obtained for mtry=120, providing an optimal interplay between mean predictive accuracy, small SD and a parsimonious nature in comparison to other mtry values tested.

Supplementary Figure S5:

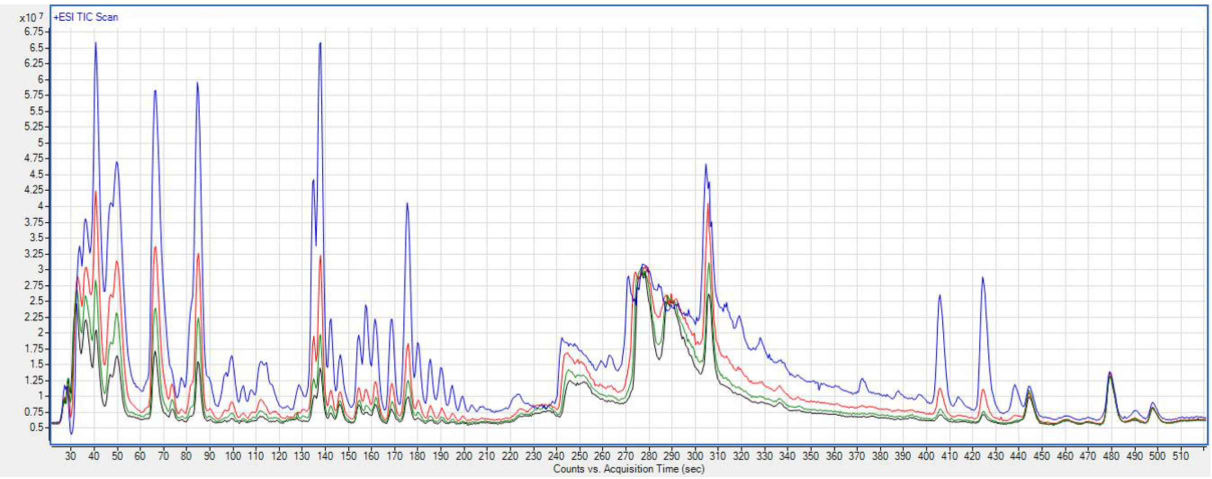

Total ion chromatogram (TIC) overlay of technical quality control dilutions, generated by pooling 4 µl of each sample. Dilutions: 1:1 (blue), 1:2 (red), 1:5 (green), and 1:10 (black). Metabolite fraction, positive ionization mode.

Supplementary Figure S6:

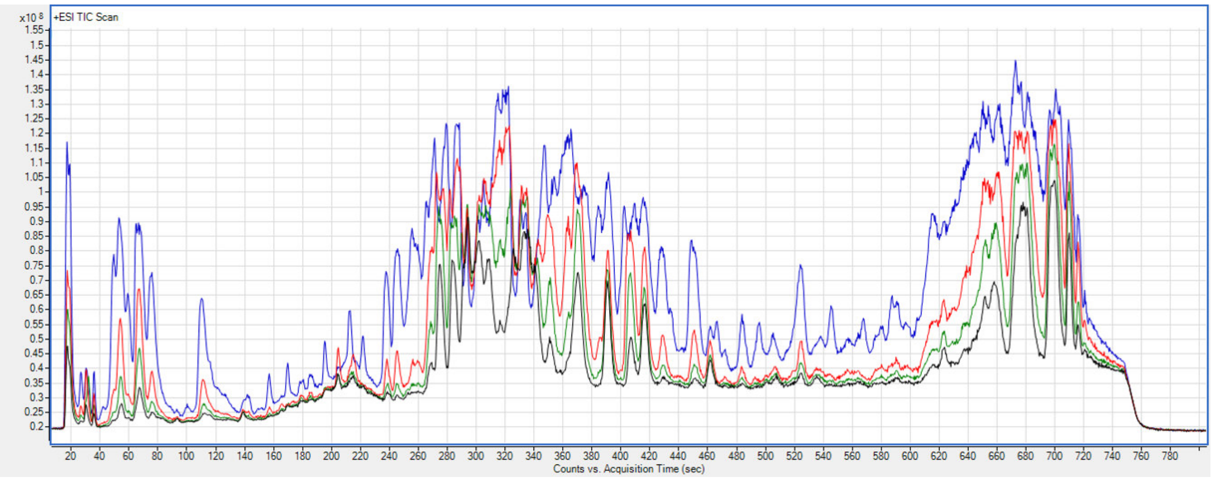

Total ion chromatogram (TIC) overlay of technical quality control dilutions, generated by pooling 4 µl of each sample. Dilutions: 1:1 (blue), 1:2 (red), 1:5 (green), and 1:10 (black). Lipid fraction, positive ionization mode.

Supplementary Information S7:

As the exposure to CM is related to a higher risk for developing psychiatric disorders later in life (20), one might assume that a concurrently present psychiatric disorder might confound the effects of CM. However, excluding CM+ women with a psychiatric disorder would result in an artificial, non-representative and possibly particularly resilient CM group. Thus, we refrained from excluding these women for our analyses. However, to assure that our results are not merely driven by the higher proportion of lifetime psychiatric diagnoses in the CM+ group, we repeated our analyses excluding these 25 women with a *SCID-I*-diagnosed lifetime psychiatric disorder from the CM group. Univariate and multivariate approaches in this reduced sample (N=34 CM+, N=46 CM-) led to similar results as for the entire sample (N=59 CM+, N=46 CM-). Six out of the eight initially significant metabolites (bilirubin IXa; PGH2-EA; ubiquinone 8; PA(O-18:0/12:0); PC(O-18:0/20:0); PI(20:0/20:4); PI(22:2/20:5); DG(18:0/20:3/0:0)) remained significant after multiple testing correction in the group comparisons (all FDR<.10; except for PGH2-EA: FDR=.52 and bilirubin IXa: FDR=.33). However, original p-values for PGH2-EA (t(44.9)=-2.23, p=.03) and bilirubin IXa (t(60.32)=-2.67, p=.01) were still significant in the group comparisons. Correlation analyses revealed the same two significant metabolites (PC(O-18:0/20:0): τ=-.37, p<.001, FDR=.008 and ubiquinone 8: τ=-.35, p<.001, FDR=.03) as for the entire CM group. Seven out of the eight initially significant metabolites were still in the first 30 top-ranked metabolites for PLS-DA and RF-CI (except for PI(20:0/20:4) in the PLS-DA and bilirubin IXa in the RF-CI).

References

1. R Core Team. *R: a language and environment for statistical computing* (R Foundation for Statistical Computing, Vienna, Austria, 2016; URL: https://www.R-project.org/).
2. Saccenti, E., Hoefsloot, H. C. J., Smilde, A. K., Westerhuis, J. A. & Hendriks, M. M. W. B. Reflections on univariate and multivariate analysis of metabolomics data. *Metabolomics* **10,** 361-374 (2014).
3. Schury, K. & Kolassa, I.-T. Biological memory of childhood maltreatment – current knowledge and recommendations for future research. *Ann. N. Y. Acad. Sci.* **1262,** 93-100 (2012).
4. Benjamini, Y. & Hochberg, Y. Controlling the False Discovery Rate: a practical and powerful approach to multiple testing. *J. R. Stat. Soc. Series B Stat. Methodol.* **57,** 289-300 (1995).
5. Noble, W. S. How does multiple testing correction work? *Nat. Biotechnol.* **27,** 1135-1137 (2009).
6. Reiner, A., Yekutieli, D. & Benjamini, Y. Identifying differentially expressed genes using false discovery rate controlling procedures. *Bioinformatics* **19,** 368-375 (2003).
7. Karabatsiakis, A. *et al.* Metabolite profiling in posttraumatic stress disorder. *J. Mol. Psychiatry* **3,** 2; 10.1186/s40303-015-0007-3 (2015).
8. Gromski, P. S. *et al.* A tutorial review: metabolomics and partial least squares-discriminant analysis – a marriage of convenience or a shotgun wedding. *Anal. Chim. Acta* **879,** 10-23 (2015).
9. Menze, B. H. *et al.* A comparison of random forest and its Gini importance with standard chemometric methods for the feature selection and classification of spectral data. *BMC Bioinformatics* **10,** 213; 10.1186/1471-2105-10-213 (2009).
10. Wold, H. Estimation of principal components and related models by iterative least squares in *Multivariate Analysis* (1966).

11. Wold, H. Path models with latent variables: The NIPALS approach in *Quantitative Sociology: International perspectives on mathematical and statistical model building* (1975).
12. Tenenhaus, M. *La regression PLS: theorie et pratique* (Editions Technip, 1998).
13. Le Cao, K.-A. *et al.* mixOmics: omics data integration project. *R package version 6.0.1* (2016).
14. Hastie, T., Tibshirani, R. & Friedman, J. *The elements of statistical learning – data mining, inference, and prediction* (Springer, 2009).
15. Chong, I.-G. & Jun, C.-H. Performance of some variable selection methods when multicollinearity is present. *Chemometr. Intell. Lab. Syst.* **78,** 103-112 (2005).
16. Hothorn, T., Buehlmann, P., Dudoit, S., Molinaro, A. & Van Der Laan, M. Survival ensembles. *Biostatistics* **7,** 355-373 (2006).
17. Strobl, C., Boulesteix, A.-L., Zeileis, A. & Hothorn, T. Bias in random forest variable importance measures: illustrations, sources and a solution. *BMC Bioinformatics* **8,** 25; 10.1186/1471-2105-8-25 (2007).
18. Strobl, C., Boulesteix, A.-L., Kneib, T., Augustin, T. & Zeileis, A. Conditional variable importance for random forests. *BMC Bioinformatics* **9,** 307; 10.1186/1471-2105-9-307 (2008).
19. Conrad, D. *et al.* Does trauma event type matter in the assessment of traumatic load? *Eur. J. Psychotraumatol.* **8,** 1344079; 10.1080/20008198.2017.1344079 (2017).
20. Li, M., D'Arcy, C. & Meng, X. Maltreatment in childhood substantially increases the risk of adult depression and anxiety in prospective cohort studies: systematic review, meta-analysis, and proportional attributable fractions. *Psychol. Med.* **46,** 717-730 (2016).

Supplementary Table S1.

List of *N* =398 detected metabolite and lipid candidates matched to METLIN Metabolomics Database.

(MM = Monoisotopic mass in Da; Rt = Retention time in min; IM = Ionization mode; eight metabolites significant in group comparisons and multivariate analyses marked in bold and italic)

Compound name	Formula	Rt	MM	METLIN ID	Class	IM
(±)-Mevalonolactone	C6 H10 O3	1,21	130,0635	44780	metabolites	negative
(24R)-1α,24-dihydroxy-22-oxavitamin D3 / (24R)-1α,24-dihydroxy-22-oxacholecalciferol	C26 H42 O4	3,63	418,3077	41996	lipids	positive
(25S)-11alpha,20,26-trihydroxyecdysone	C27 H44 O9	3,90	512,2972	57610	metabolites	negative
(Carotenoid K-G) 1'-Hydroxy-4-keto-gamma-carotene glucoside	C47 H70 O7	9,83	746,5159	41426	metabolites	positive
(S)-2,3,4,5-Tetrahydropiperidine-2-carboxylate	C6 H9 N O2	1,86	127,0633	62803	metabolites	positive
(Z)-N-(2-hydroxyethyl)hexadec-7-enamide	C18 H35 N O2	5,73	297,2665	3715	metabolites	positive
1-(O-alpha-D-glucopyranosyl)-(1,3R,29S,31R)-dotriacontanetetraol	C38 H76 O9	4,20	676,5487	46591	lipids	positive
1-(O-alpha-D-glucopyranosyl)-29-keto-(1,3R,31R)-dotriacontanetriol	C38 H74 O9	3,97	674,5352	46604	lipids	positive
1-(O-alpha-D-glucopyranosyl)-3-keto-(1,27R,29R)-triacontanetriol	C36 H70 O9	3,43	646,5022	46596	lipids	positive
1,9-Dimethyluric acid	C7 H8 N4 O3	0,78	196,0604	58078	metabolites	negative
10-Nitrooleate	C18 H33 N O4	4,62	327,2402	44828	metabolites	positive
11-amino-undecanoic acid	C11 H23 N O2	0,67	201,1708	35923	metabolites	positive
11E-octadecen-9-ynoic acid	C18 H30 O2	1,23	278,225	35214	lipids	negative
11-methyl-tridecanoic acid	C14 H28 O2	7,98	228,2089	34617	metabolites	negative
11-oxo-octadecanoic acid	C18 H34 O3	0,60	298,2505	35802	lipids	negative
12alpha-(Chloromethyl)-12-hydroxy-pregn-4-ene-3,20-dione	C22 H31 Cl O3	1,89	378,1953	70425	metabolites	positive
12-methyl-hexadecanoic acid	C17 H34 O2	2,57	270,2557	34622	lipids	negative
12R,13S-epoxy-9S-hydroxy-10E-octadecenoic acid	C18 H32 O4	4,90	312,2305	36020	metabolites	negative
13Z-hexadecenoic acid	C16 H30 O2	8,35	254,2246	34928	metabolites	negative
13Z-octadecenoic acid	C18 H34 O2	2,31	282,2561	34954	lipids	negative
15,16-dihydroxy-octadecanoic acid	C18 H36 O4	5,74	316,2606	35562	metabolites	negative
15-eicosenoic acid	C20 H38 O2	3,09	310,2868	35032	lipids	negative
15R-PGE2 methyl ester, 15-acetate	C23 H36 O6	4,46	408,2505	45956	metabolites	negative
17beta-(Acetylthio)estra-1,3,5(10)-trien-3-ol acetate	C22 H28 O3 S	3,20	372,1757	70454	metabolites	positive
17-phenyl trinor PGF2α diethyl amide	C27 H41 N O4	3,46	443,3026	45717	metabolites	positive
17ß-Hydroxy-5α-androstan-3-one sulfate	C19 H30 O5 S	4,49	370,1811	3565	metabolites	negative
17-α, 21-dihydroxy-11,20-dioxo-5-ß-pregnan-3-α-yl-ß-d-glucuronide	C27 H40 O11	2,87	540,2569	4094	metabolites	negative
19-methyl-heneicosanoic acid	C22 H44 O2	4,27	340,3327	34654	lipids	negative
1H-Indole-4-acetic acid, 2,3-dihydro-2-oxo-	C10 H9 N O3	1,06	191,0584	2373	metabolites	positive
1-Methylhypoxanthine	C6 H6 N4 O	0,91	150,0547	3779	metabolites	positive
1-Octadecanamine	C18 H39 N	7,38	269,3083	24042	metabolites	positive
2-(3'-Methylthio)propylmalic acid	C8 H14 O5 S	3,26	222,0565	64493	lipids	positive
2-(Acetamidomethylene)succinate	C7 H9 N O5	0,75	187,0479	63871	metabolites	positive
2,14-Dimethyl-octadecanoic acid	C20 H40 O2	3,58	312,3027	4330	lipids	negative
2,4-dimethyl-tetradecanoic acid	C16 H32 O2	2,05	256,2404	34599	lipids	negative
20:0 Cholesteryl ester	C47 H84 O2	9,04	726,6493	41705	lipids	negative
22-Methyl-tricosanoic acid	C24 H48 O2	5,08	368,3647	4300	lipids	negative
26,26,26-trifluoro-25-hydroxy-27-norvitamin D3 / 26,26,26-trifluoro-25-hydroxy-27-norcholecalciferol	C26 H39 F3 O2	3,66	440,2897	41965	lipids	positive

2-Aminoadenosine	C10 H14 N6 O4	1,21	282,1082	65544	metabolites	negative
2-amino-hexadecanoic acid	C16 H33 N O2	5,24	271,2513	35937	lipids	positive
2-C-Methyl-D-erythritol 4-phosphate	C5 H13 O7 P	0,57	216,0404	64013	metabolites	negative
2-ethyl-2-methyl valeric ccid	C8 H16 O2	3,05	144,1148	34581	metabolites	negative
2-Formaminobenzoylacetate	C10 H9 N O4	1,10	207,0536	63560	metabolites	negative
2-Furoic acid	C5 H4 O3	0,47	112,0158	2266	metabolites	negative
2-hydroxy-2-methyl-butyric acid	C5 H10 O3	0,67	118,0629	45847	metabolites	negative
2-hydroxypyridine	C5 H5 N O	0,81	95,0374	44708	metabolites	negative
2-keto valeric acid	C5 H8 O3	0,74	116,0469	3243	metabolites	negative
2-Methylbutyrylglycine	C7 H13 N O3	0,60	159,0895	5328	metabolites	negative
3-(2,3-Dihydroxyphenyl)propanoate	C9 H10 O4	0,67	182,0578	63526	metabolites	negative
3-(2,4-Cyclopentadien-1-ylidene)-5alpha-androstan-17beta-ol	C24 H34 O	5,32	338,26	70417	metabolites	positive
3-(4-Hydroxyphenyl)propionic acid	C9 H10 O3	1,26	166,0625	6540	metabolites	negative
3,3-Difluoro-17-methyl-5alpha-androstan-17beta-ol	C20 H32 F2 O	7,67	326,2425	70630	metabolites	positive
3,3-Difluoro-5alpha-androstan-17beta-yl acetate	C21 H32 F2 O2	4,72	354,2381	70090	metabolites	positive
3,5-dimethyl-tetradecanoic acid	C16 H32 O2	9,24	256,2407	34600	metabolites	negative
3,7,11,15-Tetramethyl-6,10,14-hexadecatrien-1-ol	C20 H36 O	3,08	292,2763	46161	lipids	positive
38:4(23Z,26Z,29Z,32Z)	C38 H68 O2	2,38	556,524	74366	metabolites	positive
3a,7b,12a-Trihydroxyoxocholanyl-Glycine	C26 H43 N O6	4,11	465,3085	58003	metabolites	negative
3-Amino-3-(4-hydroxyphenyl)propanoate	C9 H11 N O3	0,69	181,0733	6984	metabolites	positive
3b,16b-Dihydroxyandrostenone sulfate	C19 H28 O6 S	3,33	384,1607	5323	metabolites	negative
3-carboxy-4-methyl-5-propyl-2-furanpropanoic acid	C12 H16 O5	2,37	240,0999	74897	metabolites	negative
3-Deoxyvitamin D3	C27 H44	4,76	368,344	42544	lipids	positive
3-Hydroxykynurenamine	C9 H12 N2 O2	1,07	180,0895	63548	metabolites	positive
3-Methylindole	C9 H9 N	1,42	131,0732	5453	metabolites	positive
3α,12α-Dihydroxy-5ß-chol-7-en-24-oic Acid	C24 H38 O4	9,85	390,2764	42807	metabolites	positive
4,12-Dimethyl-tridecanoic acid	C15 H30 O2	8,63	242,2249	4316	metabolites	negative
4,14-Dimethyl-hexadecanoic acid	C18 H36 O2	2,98	284,2719	4327	lipids	negative
4a-peroxy-tetrahydrobiopterin	C9 H15 N5 O5	0,75	273,1066	6591	metabolites	negative
4-Benzyloxy-2'-hydroxy-3',4',5',6'-tetramethoxychalcone	C26 H26 O7	4,16	450,1686	70573	metabolites	negative
4-Carboxy-4'-sulfoazobenzene	C13 H10 N2 O5 S	0,55	306,031	66485	metabolites	negative
4-Deoxytetronic acid	C4 H6 O2	0,47	86,0371	5533	metabolites	negative
4-Hydroxy-4-(3-pyridyl)-butanoic acid	C9 H11 N O3	0,65	181,0733	6015	metabolites	negative
4-Hydroxybenzaldehyde	C7 H6 O2	0,69	122,0361	62451	metabolites	positive
4R-aminopentanoic acid	C5 H11 N O2	0,62	117,0792	35940	metabolites	negative
4-Sulfobenzyl alcohol	C7 H8 O4 S	2,13	188,0144	66489	metabolites	negative
4Z,15E-Bilirubin IXa	C33 H36 N4 O6	6,71	584,262	5445	metabolites	negative
5-Acetylamino-6-amino-3-methyluracil	C7 H10 N4 O3	0,72	198,0759	58239	metabolites	negative
5-alpha-Dihydrotestosterone glucuronide	C25 H38 O8	3,91	466,2563	57959	metabolites	negative
5-Aminopentanoic acid	C5 H11 N O2	0,55	117,0788	6902	metabolites	positive
5'-Deoxy-5'-(methylthio)adenosine	C11 H15 N5 O3 S	1,45	297,0895	3425	metabolites	positive
5-methyl-octanoic acid	C9 H18 O2	4,28	158,1309	34665	metabolites	negative

5S-HETE di-endoperoxide	C20 H34 O8	7,99	402,2252	74966	metabolites	positive
5ß-Cholanic acid-3α, 12α-diol N-(2-sulphoethyl)-amide	C26 H45 N O6 S	3,55	499,2977	44688	metabolites	positive
5-undecenoic acid	C11 H20 O2	4,93	184,1463	35261	metabolites	negative
6E,9E-octadecadienoic acid	C18 H32 O2	1,68	280,2403	34994	lipids	negative
7ß,12α-Dihydroxy-5α-cholan-24-oic Acid	C24 H40 O4	5,43	392,2921	42661	metabolites	negative
9E-tetradecenoic acid	C14 H26 O2	7,07	226,1933	34915	metabolites	negative
Adenine	C5 H5 N5	1,06	135,0544	85	metabolites	negative
AFMK	C13 H16 N2 O4	1,92	264,111	44788	metabolites	positive
Alpha-N-Phenylacetyl-L-glutamine	C13 H16 N2 O4	1,38	264,1116	58397	metabolites	negative
Arabinosylhypoxanthine	C10 H12 N4 O5	0,73	268,0809	3022	metabolites	negative
Aryl beta-D-glucoside	C12 H16 O6	0,81	256,0955	65906	metabolites	negative
Ascorbate 2-sulfate	C6 H8 O9 S	0,47	255,9891	65843	metabolites	negative
b-D-Glucopyranosiduronic acid	C15 H21 N O8	0,67	343,1264	2024	metabolites	negative
Behenoyl-EA	C24 H49 N O2	2,31	383,3751	3727	lipids	positive
Benzaldehyde	C7 H6 O	1,11	106,0414	58358	metabolites	positive
Bis(glutathionyl)spermine	C30 H56 N10 O10 S2	2,59	780,3594	63643	metabolites	positive
Butyric acid	C4 H8 O2	1,23	88,0522	107	metabolites	positive
C16 Sphinganine	C16 H35 N O2	5,11	273,267	41556	metabolites	positive
C16 Sulfatide	C40 H77 N O11 S	3,83	779,5189	41623	lipids	negative
C16-OH Sulfatide	C40 H77 N O12 S	5,00	795,5179	41624	lipids	positive
C17 Sphinganine	C17 H37 N O2	5,15	287,282	41558	metabolites	positive
C18-OH Sulfatide	C42 H81 N O12 S	5,68	823,5518	41625	lipids	positive
Cer(d16:1/23:0)	C39 H77 N O3	7,31	653,5947	83716	lipids	negative
Cer(d18:0/14:0)	C32 H65 N O3	4,35	511,4953	53968	lipids	positive
Cer(d18:0/16:0)	C34 H69 N O3	5,20	539,5268	41565	lipids	positive
Cer(d18:0/17:0)	C35 H71 N O3	7,05	553,5425	83730	lipids	positive
Cer(d18:0/18:0)	C36 H73 N O3	6,02	567,5581	41566	lipids	positive
Cer(d18:0/20:0)	C38 H77 N O3	6,94	595,5893	41567	lipids	positive
Cer(d18:0/22:0)	C40 H81 N O3	7,84	623,6175	41568	lipids	positive
Cer(d18:1/17:0)	C35 H69 N O3	5,74	597,5317	83719	lipids	negative
Cer(d18:2/18:1)	C36 H67 N O3	5,20	561,5095	83724	lipids	positive
Cer(d18:2/23:0)	C41 H79 N O3	7,85	633,6048	83729	lipids	positive
Cer(t18:0/26:0(2-OH))	C44 H89 N O5	8,97	711,6708	53973	lipids	negative
Ceramide (d18:1/22:0)	C40 H79 N O3	8,03	621,6068	7205	lipids	negative
Ceramide (d18:1/24:0)	C42 H83 N O3	8,71	649,6366	7209	lipids	negative
Ceramide (d18:1/24:1(15Z))	C42 H81 N O3	8,03	647,6205	7206	lipids	negative
Ceramide (d18:1/25:0)	C43 H85 N O3	8,71	709,6582	7210	lipids	negative
Ceramide (d18:1/26:0)	C44 H87 N O3	8,93	723,6717	7208	lipids	negative
CerP(d18:1/16:0)	C34 H68 N O6 P	4,55	617,4772	41575	lipids	negative
CerP(d18:1/24:1(15Z))	C42 H82 N O6 P	6,74	727,5866	41580	lipids	negative
Cholesteryl stearate	C45 H80 O2	8,37	698,6189	5898	lipids	negative
CL(1'-[22:1(13Z)/22:1(13Z)],3'-[22:1(13Z)/14:1(9Z)])[rac]	C89 H166 O17 P2	5,53	1569,1633	40955	lipids	positive

Coenzyme Q1	C14 H18 O4	5,43	250,1202	45118	metabolites	positive
Cortisol	C21 H30 O5	3,71	362,2088	272	metabolites	positive
Cortolone-3-glucuronide	C27 H42 O11	2,69	542,2719	61643	metabolites	negative
Creatine	C4 H9 N3 O2	0,56	131,0693	7	metabolites	positive
Creatinine	C4 H7 N3 O	0,55	113,0587	8	metabolites	positive
Cytosine	C4 H5 N3 O	1,04	111,0433	283	metabolites	positive
Dehydroascorbic acid	C6 H6 O6	0,47	174,017	342	metabolites	negative
Deoxyguanosine	C10 H13 N5 O4	1,06	267,097	3395	metabolites	negative
DG(14:0/18:3(9Z,12Z,15Z)/0:0)	C35 H62 O5	5,94	562,4589	58653	lipids	positive
DG(16:0e/18:0/0:0)	C37 H74 O4	7,66	628,5643	62000	lipids	negative
DG(17:1(9Z)/17:2(9Z,12Z)/0:0)[iso2]	C37 H66 O5	7,22	590,4876	4279	lipids	positive
DG(18:0/18:3(6Z,9Z,12Z)/0:0)	C39 H70 O5	7,96	618,5194	58757	lipids	positive
DG(18:0/20:3(5Z,8Z,11Z)/0:0)	C41 H74 O5	8,65	646,5508	58759	lipids	positive
DG(18:1(11Z)/18:3(9Z,12Z,15Z)/0:0)	C39 H68 O5	7,28	616,5045	58774	lipids	positive
DG(18:2(9Z,12Z)/18:3(6Z,9Z,12Z)/0:0)	C39 H66 O5	6,70	614,4899	58814	lipids	positive
DG(18:4(6Z,9Z,12Z,15Z)/20:1(11Z)/0:0)	C41 H70 O5	7,38	642,5195	58879	lipids	positive
DG(18:4(6Z,9Z,12Z,15Z)/20:2(11Z,14Z)/0:0)	C41 H68 O5	6,83	640,5037	58880	lipids	positive
DG(22:4(7Z,10Z,13Z,16Z)/22:4(7Z,10Z,13Z,16Z)/0:0)	C47 H76 O5	8,94	720,5697	4673	lipids	positive
DG(P-14:0/18:1(9Z))	C35 H66 O4	3,01	596,4986	4697	lipids	negative
D-Proline	C5 H9 N O2	0,56	115,0635	6923	metabolites	positive
D-α-Hydroxyglutaric acid	C5 H8 O5	8,30	148,0371	45075	metabolites	positive
Eicosanoyl-EA	C22 H45 N O2	7,34	355,3442	3723	metabolites	positive
Elaidamide	C18 H35 N O	8,67	281,2712	36674	metabolites	positive
Etn-1-P-Cer(d14:1/18:0)	C38 H77 N2 O6 P	4,27	688,5509	41602	lipids	positive
farnesyl triphosphate	C15 H29 O10 P3	1,42	462,0987	53847	metabolites	positive
Flupenthixol-O-glucuronide	C29 H34 F3 N3 O7 S	4,78	625,2071	2773	lipids	positive
FMC-5(d18:1/22:0)	C56 H99 N O13	6,73	993,708	83799	lipids	positive
Galbeta1-4Glcbeta-Cer(d18:1/26:0)	C56 H107 N O13	6,01	1001,7708	53995	lipids	positive
GalCer(d18:1/23:0)	C47 H91 N O8	7,12	843,6781	83835	lipids	negative
GlcCer(d18:1/24:0)	C48 H93 N O8	7,83	811,6885	41612	lipids	negative
Glucosylceramide (d18:1/16:0)	C40 H77 N O8	4,92	699,5628	7224	lipids	negative
Glucosylceramide (d18:1/22:0)	C46 H89 N O8	7,12	783,6573	7227	lipids	negative
Glucosylceramide (d18:1/24:1(15Z))	C48 H91 N O8	7,12	809,6727	7228	lipids	negative
Glucosylceramide (d18:1/25:0)	C49 H95 N O8	7,83	871,7094	7232	lipids	negative
Glutamylphenylalanine	C14 H18 N2 O5	1,70	294,121	5573	metabolites	positive
Glycerophospho-N-Arachidonoyl Ethanolamine	C25 H44 N O7 P	6,73	501,2846	46715	metabolites	positive
Glycerophospho-N-Oleoyl Ethanolamine	C23 H46 N O7 P	7,27	479,3	45335	metabolites	positive
Glycine, N-[(3a,5b,7a)-3-hydroxy-24-oxo-7-(sulfooxy)cholan-24-yl]-	C26 H43 N O8 S	2,38	529,2731	6701	metabolites	positive
Glycodeoxycholate	C26 H43 N O5	4,64	449,3144	7000	metabolites	negative
Glycoursodeoxycholic acid	C26 H43 N O5	5,48	449,3135	5676	metabolites	positive
GPInsP[3'](17:0/20:4(5Z,8Z,11Z,14Z))	C46 H82 O16 P2	2,30	952,5064	40892	metabolites	positive
Hypoxanthine	C5 H4 N4 O	0,71	136,0385	83	metabolites	positive

Indole	C8 H7 N	1,30	117,0579	286	metabolites	negative
Indoleacrylic acid	C11 H9 N O2	1,65	187,0635	5702	metabolites	positive
Indoxylsulfuric acid	C8 H7 N O4 S	1,58	213,0098	253	metabolites	negative
Isoguanosine	C10 H13 N5 O5	0,74	283,092	66971	metabolites	negative
Kynurenine	C10 H12 N2 O3	1,06	208,0845	72	metabolites	positive
LacCer(d18:0/16:0)	C46 H89 N O13	6,32	863,6362	83818	lipids	positive
LacCer(d18:0/26:0)	C56 H109 N O13	6,51	1003,7862	83823	lipids	positive
Lactosylceramide (d18:1/16:0)	C46 H87 N O13	4,59	861,616	7125	lipids	positive
Lactosylceramide (d18:1/24:1(15Z))	C54 H101 N O13	6,73	971,724	7129	lipids	positive
Lanthionine ketimine	C6 H7 N O4 S	1,01	189,0105	7085	metabolites	negative
L-Arginine	C6 H14 N4 O2	0,47	174,1115	13	metabolites	positive
Lauric acid	C12 H24 O2	6,59	200,1783	357	metabolites	negative
Levulinic Acid, 3-Benzylidenyl-	C12 H12 O3	7,40	204,0784	44382	metabolites	positive
L-Glutamic acid n-butyl ester	9 H17 N O4	0,83	203,1159	3544	metabolites	negative
L-Homocysteic acid	C4 H9 N O5 S	0,62	183,0202	6545	metabolites	negative
Linoleamide	C18 H33 N O	5,73	279,2558	43435	metabolites	positive
L-Isoleucine	C6 H13 N O2	0,84	131,0947	23	metabolites	positive
lithocholic acid sulfate	C24 H40 O6 S	4,41	456,2538	210	metabolites	negative
L-Phenylalanine	C9 H11 N O2	1,11	165,0794	28	metabolites	positive
LysoPE(0:0/15:0)	C20 H42 N O7 P	5,34	439,2691	2260	metabolites	positive
LysoPE(0:0/18:2(9Z,12Z))	C23 H44 N O7 P	7,27	477,2852	62265	metabolites	negative
LysoPE(0:0/20:0)	C25 H52 N O7 P	7,64	555,3536	62269	metabolites	negative
LysoPE(0:0/20:0) 7.4149957	C25 H52 N O7 P	7,41	509,3466	62269	metabolites	positive
LysoPE(0:0/20:1(11Z))	C25 H50 N O7 P	7,89	507,3318	62270	metabolites	negative
LysoPE(0:0/20:2(11Z,14Z))	C25 H48 N O7 P	7,31	505,3162	62271	metabolites	negative
LysoPE(0:0/22:0)	C27 H56 N O7 P	1,76	583,3842	62278	lipids	negative
LysoPE(0:0/22:6(4Z,7Z,10Z,13Z,16Z,19Z))	C27 H44 N O7 P	6,71	525,2841	62284	metabolites	positive
LysoPE(20:1(11Z)/0:0)	C25 H50 N O7 P	0,99	507,3326	62297	lipids	positive
Mesaconic acid	C5 H6 O4	0,47	130,0263	4130	metabolites	negative
Metalaxyl	C15 H21 N O4	2,06	279,1471	68712	metabolites	positive
methyl 8-[2-(2-formyl-vinyl)-3-hydroxy-5-oxo-cyclopentyl]-octanoate	C17 H26 O5	5,48	310,1777	35518	metabolites	positive
Methyl N-butyrylglycine	C7 H13 N O3	0,92	159,0885	5654	metabolites	positive
Methylthiobenzoic acid	C8 H8 O2 S	3,25	214,0307	2538	metabolites	negative
MID41949:(6RS)-22-oxo-23,24,25,26,27-pentanorvitamin D3 6,19-sulfur dioxide adduct / (6RS)-22-oxo-23	C22 H32 O4 S	2,13	392,2022	41949	metabolites	positive
N-(2'-(4-benzenesulfonamide)-ethyl) arachidonoyl amine	C28 H42 N2 O3 S	0,31	486,2914	36721	lipids	positive
N-(3S-hydroxydecanoyl)-L-serine	C13 H25 N O5	1,48	275,1728	45741	metabolites	positive
N4-Acetylcytidine	C11 H15 N3 O6	1,04	285,0962	58335	metabolites	positive
N-Acetyl-L-phenylalanine	C11 H13 N O3	1,05	207,0894	34536	metabolites	negative
N-acetyl-LTE4	C25 H39 N O6 S	2,81	527,2546	36239	metabolites	negative
NAc-FnorLRF-amide	C32 H46 N8 O5	7,33	622,3598	69127	lipids	positive
Narasin	C43 H72 O11	3,97	764,5061	43270	lipids	positive
N-Cyclohexanecarbonylpentadecylamine	C22 H43 N O	9,93	337,3342	45078	metabolites	positive

N-docosahexaenoyl GABA	C26 H39 N O3	5,32	413,2923	75487	metabolites	positive
N-Formylmethionine	C6 H11 N O3 S	0,74	177,0464	65914	metabolites	negative
N-Hydroxymethylnicotinamide	C7 H8 N2 O2	0,76	152,058	44230	metabolites	positive
N-Isopropylterephthalaldehydamide	C11 H13 N O2	1,71	237,0999	2109	metabolites	negative
N-methyl arachidonoyl amine	C21 H35 N O	5,13	317,2723	36679	metabolites	positive
N-Methylethanolamine phosphate	C3 H10 N O4 P	0,51	155,0344	63347	metabolites	positive
N-Oleoyl-L-Serine	C21 H39 N O4	5,86	369,2876	45444	metabolites	positive
N-palmitoyl glutamic acid	C21 H39 N O5	5,15	385,2821	75469	metabolites	positive
N-palmitoyl serine	C19 H37 N O4	5,50	343,2721	75483	metabolites	positive
N-Palmitoylsphingosine	C34 H67 N O3	5,74	537,513	5756	lipids	negative
N-stearoyl serine	C21 H41 N O4	6,27	371,3029	75484	metabolites	positive
O-Benzyl-L-Serine	C10 H13 N O3	1,20	195,0893	44267	metabolites	positive
Oleoyl Ethyl Amide	C20 H39 N O	6,94	309,3033	44905	lipids	positive
Oxalosuccinic acid	C6 H6 O7	2,13	190,0117	3326	metabolites	negative
PA(18:0/18:4(6Z,9Z,12Z,15Z))	C39 H69 O8 P	4,86	696,471	82052	lipids	negative
PA(18:1(9Z)/16:1(9Z))	C37 H69 O8 P	4,94	672,4712	82047	lipids	negative
PA(20:0/14:1(9Z))	C37 H71 O8 P	5,45	674,4865	81675	lipids	negative
PA(20:0/18:0)	C41 H81 O8 P	9,60	732,5671	82120	lipids	positive
PA(20:5(5Z,8Z,11Z,14Z,17Z)/20:4(5Z,8Z,11Z,14Z))	C43 H67 O8 P	4,47	742,4539	81835	lipids	positive
PA(20:5(5Z,8Z,11Z,14Z,17Z)/22:6(4Z,7Z,10Z,13Z,16Z,19Z))	C45 H67 O8 P	9,24	766,4548	82021	metabolites	negative
PA(22:0/18:1(9Z))	C43 H83 O8 P	9,68	758,5816	82113	lipids	positive
PA(22:0/20:2(11Z,14Z))	C45 H85 O8 P	9,78	784,5984	81882	lipids	positive
PA(22:1(11Z)/20:0)	C45 H87 O8 P	10,22	786,615	81909	lipids	positive
PA(22:2(13Z,16Z)/21:0)	C46 H87 O8 P	10,05	798,6136	81946	lipids	positive
PA(22:6(4Z,7Z,10Z,13Z,16Z,19Z)/0:0)	C25 H39 O7 P	0,90	482,241	82338	lipids	positive
PA(22:6(4Z,7Z,10Z,13Z,16Z,19Z)/22:1(11Z))	C47 H79 O8 P	6,51	802,5526	82008	lipids	positive
PA(O-16:0/19:0)	C38 H77 O7 P	6,12	676,54	82224	lipids	negative
PA(O-18:0/12:0)	C33 H67 O7 P	7,20	606,4624	82165	lipids	positive
PA(O-18:0/13:0)	C34 H69 O7 P	4,86	620,4769	82166	lipids	positive
PA(O-18:0/19:0)	C40 H81 O7 P	6,91	704,5724	82179	lipids	negative
PA(P-16:0/18:2(9Z,12Z))	C37 H69 O7 P	6,83	656,4781	82328	lipids	positive
PA(P-18:0/14:1(9Z))	C35 H67 O7 P	6,70	630,463	82271	lipids	positive
PA(P-20:0/14:1(9Z))	C37 H71 O7 P	7,38	658,494	82299	lipids	positive
p-Acetaminobenzoic acid	C9 H9 N O3	1,89	179,0584	2830	metabolites	positive
Palmitic amide	C16 H33 N O	2,13	255,2556	62905	lipids	positive
Pantothenic Acid	C9 H17 N O5	1,21	219,1109	241	metabolites	positive
PC(13:0/0:0)[U]	C21 H44 N O7 P	7,04	453,2843	40776	metabolites	positive
PC(13:0/20:4(5Z,8Z,11Z,14Z))	C41 H74 N O8 P	5,07	739,516	60447	lipids	positive
PC(14:0/0:0)[U]	C22 H46 N O7 P	6,21	467,3011	77696	metabolites	positive
PC(16:0/14:0)[U]	C38 H76 N O8 P	4,70	705,531	60417	lipids	positive
PC(16:1(9Z)/0:0)[U]	C24 H48 N O7 P	6,47	493,317	77685	metabolites	positive
PC(17:0/11:0)	C36 H72 N O8 P	4,09	677,4971	60333	lipids	positive

PC(17:0/15:0)	C40 H80 N O8 P	5,35	733,5623	40745	lipids	positive
PC(17:0/22:6(4Z,7Z,10Z,13Z,16Z,19Z))	C47 H82 N O8 P	5,05	819,5768	60983	lipids	positive
PC(17:1(9Z)/13:0)	C38 H74 N O8 P	4,20	703,515	76859	lipids	positive
PC(17:1(9Z)/20:5(5Z,8Z,11Z,14Z,17Z))	C45 H78 N O8 P	4,42	791,5451	60727	lipids	positive
PC(17:2(9Z,12Z)/16:0)	C41 H78 N O8	4,63	743,546	60287	lipids	positive
PC(18:0/17:1(9Z))	C43 H84 N O8 P	5,82	773,5924	60449	lipids	positive
PC(18:0/P-16:0)	C42 H84 N O7 P	5,91	745,5967	77628	lipids	positive
PC(18:1(11Z)/20:2(11Z,14Z))	C46 H86 N O8 P	5,85	811,6091	77244	lipids	positive
PC(18:1(9Z)/18:0)[U]	C44 H86 N O8 P	6,18	787,6103	60358	lipids	positive
PC(18:2(2Z,4Z)/18:2(2Z,4Z))	C44 H81 N O8 P	5,46	781,5613	39665	lipids	positive
PC(18:2(9Z,12Z)/17:0)	C43 H82 N O8 P	5,30	771,5771	40722	lipids	positive
PC(18:2(9Z,12Z)/18:2(9Z,12Z))[S]	C44 H80 N O8 P	4,58	781,5599	76795	lipids	positive
PC(18:3(6Z,9Z,12Z)/20:2(11Z,14Z))	C46 H82 N O8 P	5,12	807,576	77246	lipids	positive
PC(18:4(6Z,9Z,12Z,15Z)/18:1(9Z))	C44 H78 N O8 P	4,19	779,5459	77346	lipids	positive
PC(18:4(6Z,9Z,12Z,15Z)/20:4(8Z,11Z,14Z,17Z))	C46 H77 N O8 P	4,47	801,5277	59693	lipids	positive
PC(18:4(6Z,9Z,12Z,15Z)/22:2(13Z,16Z))	C48 H84 N O8 P	5,39	833,5926	3866	lipids	positive
PC(19:0/12:0)	C39 H78 N O8 P	5,01	719,5473	60418	lipids	positive
PC(19:0/14:1(9Z))	C41 H80 N O8 P	5,12	745,5619	40718	lipids	positive
PC(19:0/18:4(6Z,9Z,12Z,15Z))	C45 H82 N O8 P	5,21	795,5771	60725	lipids	positive
PC(2:0/O-16:0)[U]	C26 H54 N O7 P	1,86	523,3631	77692	lipids	positive
PC(20:0/20:4(8Z,11Z,14Z,17Z))	C48 H88 N O8 P	6,07	837,6241	77362	lipids	positive
PC(O-14:0/2:0)	C24 H50 N O7 P	7,08	495,3323	77694	metabolites	positive
PC(O-16:0/14:0)	C38 H78 N O7 P	5,09	691,551	77581	lipids	positive
PC(O-16:2(9E,10E)/0:0)[U]	C24 H48 N O6 P	1,12	477,3215	46721	lipids	positive
PC(O-18:0/20:0)	C46 H94 N O7 P	8,65	803,6784	77564	lipids	positive
PC(P-16:0/14:0)	C38 H76 N O7 P	5,00	689,5347	77641	lipids	positive
PC(P-20:0/22:4(7Z,10Z,13Z,16Z))	C50 H93 N O7 P	6,77	832,6341	76564	lipids	positive
p-cresol	C7 H8 O	2,13	108,0573	4236	metabolites	negative
PE(15:0/24:1(15Z))	C46 H84 N O8 P	6,18	809,5905	77354	lipids	positive
PE(16:1(9Z)/20:3(8Z,11Z,14Z))	C43 H78 N O8 P	5,74	767,5456	60398	lipids	negative
PE(16:1(9Z)/P-18:1(9Z))	C39 H74 N O7 P	5,51	699,5199	60521	lipids	positive
PE(17:1(9Z)/18:0)	C42 H76 N O8 P	4,79	753,5285	76960	lipids	positive
PE(17:2(9Z,12Z)/20:0)	C42 H80 N O8 P	4,94	757,5617	76844	lipids	negative
PE(18:0/0:0)	C23 H48 N O7 P	7,64	481,316	40775	metabolites	negative
PE(18:0/16:1(9Z))	C39 H76 N O8 P	5,64	717,5285	60419	lipids	negative
PE(18:1(11Z)/P-18:0)	C41 H80 N O7 P	6,75	729,5692	60466	lipids	negative
PE(18:3(6Z,9Z,12Z)/18:0)	C41 H76 N O8 P	5,22	741,5296	60530	lipids	negative
PE(18:3(6Z,9Z,12Z)/19:0)	C44 H76 N O8 P	4,54	777,5284	77374	lipids	positive
PE(19:0/20:3(8Z,11Z,14Z))	C46 H80 N O8 P	5,14	805,5594	77027	lipids	positive
PE(19:0/20:4(5Z,8Z,11Z,14Z))	C46 H78 N O8 P	4,58	803,5427	77384	lipids	positive
PE(19:1(9Z)/15:1(9Z))	C39 H74 N O8 P	5,11	715,5135	77033	lipids	negative
PE(20:1(11Z)/18:0)	C43 H84 N O8 P	6,18	773,5922	60651	lipids	negative

PE(20:1(11Z)/18:2(9Z,12Z))	C43 H80 N O8 P	6,03	769,5586	60654	lipids	negative
PE(20:4(8Z,11Z,14Z,17Z)/P-16:0)	C41 H74 N O7 P	5,36	723,5183	60840	lipids	negative
PE(21:0/17:0)[U]	C43 H86 N O8 P	6,06	821,6139	40605	lipids	negative
PE(22:4(7Z,10Z,13Z,16Z)/P-16:0)	C43 H78 N O7 P	6,14	751,5517	60777	lipids	positive
PE(22:5(7Z,10Z,13Z,16Z,19Z)/P-16:0)	C43 H76 N O7 P	5,45	749,5341	61064	lipids	negative
PE(22:6(4Z,7Z,10Z,13Z,16Z,19Z)/P-18:1(9Z))	C45 H76 N O7 P	5,28	773,5344	61099	lipids	negative
PE(O-16:0/17:0)	C40 H76 N O7 P	5,09	713,531	77622	lipids	positive
PE(O-16:0/19:0)	C40 H82 N O7 P	5,81	719,5818	77576	lipids	positive
PE(O-20:0/15:0)	C42 H80 N O7 P	5,82	741,5644	77650	lipids	positive
PE(P-18:0/18:2(9Z,12Z))	C41 H78 N O7 P	6,20	727,5504	62181	lipids	negative
PE(P-18:0/18:3(9Z,12Z,15Z))	C41 H76 N O7 P	5,60	725,5358	60774	lipids	positive
PE(P-18:0/19:1(9Z))	C42 H82 N O7 P	5,80	743,5803	77649	lipids	positive
PE-NMe(17:0/17:0)[U]	C42 H78 N O8 P	5,35	755,5436	76931	lipids	positive
PEP-16:0/18:1(11Z))	C39 H76 N O7 P	6,01	701,5345	62152	lipids	negative
PG(16:0/13:0)	C35 H69 O10 P	4,85	680,4655	79017	lipids	positive
PG(17:0/18:4(6Z,9Z,12Z,15Z))	C41 H73 O10 P	5,45	756,4905	79061	lipids	negative
PG(17:2(9Z,12Z)/20:2(11Z,14Z))	C43 H77 O10 P	6,17	784,522	79123	lipids	negative
PG(19:1(9Z)/18:4(6Z,9Z,12Z,15Z))	C43 H75 O10 P	5,64	782,5063	79321	lipids	negative
PG(O-18:0/22:0)	C46 H93 O9 P	9,97	820,6551	79895	lipids	positive
PG(P-20:0/22:0)	C48 H95 O9 P	7,12	846,6707	79986	lipids	negative
PGH2-EA	C23 H39 N O4	5,11	393,2867	74984	metabolites	positive
Phenyl sulfate	C6 H6 O4 S	1,43	173,9991	1828	metabolites	negative
PI(16:0/20:5(5Z,8Z,11Z,14Z,17Z))	C45 H77 O13 P	4,28	856,5071	80896	lipids	positive
PI(17:1(9Z)/17:1(9Z))	C43 H79 O13 P	4,04	834,5233	80884	lipids	negative
PI(18:0/0:0)	C27 H53 O12 P	7,77	600,3271	46746	metabolites	negative
PI(18:0/18:2(9Z,12Z))	C45 H83 O13 P	4,60	862,5563	80978	lipids	negative
PI(18:1(9Z)/20:3(8Z,11Z,14Z))	C47 H83 O13 P	4,54	886,5572	61228	lipids	negative
PI(18:2(9Z,12Z)/0:0)	C27 H49 O12 P	6,64	596,2955	81175	metabolites	negative
PI(20:0/16:1(9Z))	C45 H85 O13 P	5,04	864,571	80504	lipids	negative
PI(20:0/20:4(5Z,8Z,11Z,14Z))	C49 H87 O13 P	3,92	914,5876	80853	lipids	negative
PI(20:1(11Z)/14:0)	C43 H81 O13 P	4,44	836,5401	80520	lipids	negative
PI(20:1(11Z)/17:1(9Z))	C46 H85 O13 P	9,30	876,5759	80527	metabolites	positive
PI(20:3(5Z,8Z,11Z)/18:2(9Z,12Z))	C47 H81 O13 P	4,07	884,5391	61268	lipids	negative
PI(22:2(13Z,16Z)/16:1(9Z))	C47 H85 O13 P	4,76	888,5711	80754	lipids	negative
PI(22:2(13Z,16Z)/18:1(9Z))	C49 H89 O13 P	3,98	916,603	80759	lipids	negative
PI(22:2(13Z,16Z)/18:3(9Z,12Z,15Z))	C49 H85 O13 P	4,56	912,5764	80762	lipids	negative
PI(22:2(13Z,16Z)/20:5(5Z,8Z,11Z,14Z,17Z))	C51 H85 O13 P	3,94	936,5721	80771	lipids	positive
PI(22:4(7Z,10Z,13Z,16Z)/20:2(11Z,14Z))	C51 H87 O13 P	3,99	938,5888	80799	lipids	positive
PI(22:6(4Z,7Z,10Z,13Z,16Z,19Z)/22:2(13Z,16Z))	C53 H87 O13 P	4,55	1008,5951	80835	lipids	negative
PI(P-16:0/22:6(4Z,7Z,10Z,13Z,16Z,19Z))	C47 H79 O12 P	5,48	866,5324	81170	lipids	positive
p-Nitroglutethimide	C13 H14 N2 O4	3,21	262,0946	948	metabolites	positive
Pregnanolone sulfate	C21 H34 O5 S	4,39	398,213	3557	metabolites	negative

protoporphyrin IX	C34 H36 N4 O4	2,51	564,2746	4158	metabolites	positive
PS(13:0/22:0)	C41 H80 N O10 P	4,79	777,5498	77752	lipids	negative
PS(15:0/22:0)	C43 H84 N O10 P	5,45	805,5831	77820	lipids	negative
PS(16:0/13:0)	C35 H68 N O10 P	2,20	693,4577	77856	lipids	positive
PS(16:0/22:1(11Z))	C44 H84 N O10 P	4,94	817,5821	77863	lipids	negative
PS(17:0/22:1(11Z))	C45 H86 N O10 P	5,64	831,5993	77909	lipids	negative
PS(17:2(9Z,12Z)/22:1(11Z))	C45 H82 N O10 P	4,56	827,5641	77968	lipids	negative
PS(18:0/22:0)	C46 H90 N O10 P	6,18	847,63	78639	lipids	negative
PS(18:0/22:2(13Z,16Z))	C46 H86 N O10 P	5,13	843,5988	77985	lipids	negative
PS(18:1(9Z)/19:0)	C43 H82 N O10 P	4,62	803,5645	77996	lipids	negative
PS(18:1(9Z)/22:2(13Z,16Z))	C46 H84 N O10 P	4,57	841,5804	78002	lipids	negative
PS(18:2(9Z,12Z)/20:0)	C44 H82 N O10 P	4,50	815,5654	78020	lipids	negative
PS(19:0/0:0)	C25 H50 N O9 P	0,29	539,3224	78855	lipids	positive
PS(19:1(9Z)/21:0)	C46 H88 N O10 P	5,64	845,6148	78169	lipids	negative
PS(19:1(9Z)/22:2(13Z,16Z))	C47 H86 N O10 P	5,20	855,5954	78172	lipids	negative
PS(20:0/19:0)	C45 H88 N O10 P	6,18	833,6135	78184	lipids	negative
PS(20:1(11Z)/16:0)	C42 H80 N O10 P	4,31	789,5493	78198	lipids	negative
PS(20:2(11Z,14Z)/22:2(13Z,16Z))	C48 H86 N O10 P	4,91	867,5973	78250	lipids	negative
PS(20:3(8Z,11Z,14Z)/19:1(9Z))	C45 H80 N O10 P	5,83	825,5475	78271	lipids	negative
PS(20:3(8Z,11Z,14Z)/20:1(11Z))	C46 H82 N O10 P	4,45	839,5667	78273	lipids	negative
PS(20:3(8Z,11Z,14Z)/22:2(13Z,16Z))	C48 H84 N O10 P	4,48	865,58	78281	lipids	negative
PS(20:4(5Z,8Z,11Z,14Z)/19:1(9Z))	C45 H78 N O10 P	5,35	823,5337	78301	lipids	positive
PS(21:0/17:0)	C44 H86 N O10 P	5,45	819,5981	78346	lipids	negative
PS(21:0/20:3(8Z,11Z,14Z))	C47 H86 N O10 P	5,45	901,601	78359	lipids	negative
PS(21:0/20:4(5Z,8Z,11Z,14Z))	C47 H84 N O10 P	4,94	853,5805	78360	lipids	negative
PS(22:0/0:0)	C28 H56 N O9 P	7,89	581,3689	78852	metabolites	negative
PS(22:0/22:4(7Z,10Z,13Z,16Z))	C50 H90 N O10 P	5,59	895,6268	78388	lipids	negative
PS(22:1(11Z)/0:0)	C28 H54 N O9 P	7,31	579,353	78850	metabolites	negative
PS(22:1(11Z)/20:3(8Z,11Z,14Z))	C48 H86 N O10 P	5,64	913,6027	78411	lipids	negative
PS(22:2(13Z,16Z)/18:1(9Z))	C46 H84 N O10 P	5,45	887,5864	78432	lipids	negative
PS(22:2(13Z,16Z)/18:2(9Z,12Z))	C46 H82 N O10 P	4,94	885,5724	78433	lipids	negative
PS(22:4(7Z,10Z,13Z,16Z)/21:0)	C49 H88 N O10 P	5,64	927,6178	78476	lipids	negative
PS(22:4(7Z,10Z,13Z,16Z)/22:1(11Z))	C50 H88 N O10 P	5,38	893,6132	78478	lipids	negative
PS(22:6(4Z,7Z,10Z,13Z,16Z,19Z)/22:0)	C50 H86 N O10 P	4,80	891,5949	78506	lipids	negative
PS(O-18:0/0:0)	C24 H50 N O8 P	4,94	511,3271	78862	metabolites	positive
PS(O-20:0/0:0)	C26 H54 N O8 P	5,65	539,3578	78861	metabolites	positive
PS(O-20:0/18:0)	C44 H88 N O9 P	5,91	805,6208	78698	lipids	negative
PS(O-20:0/20:4(5Z,8Z,11Z,14Z))	C46 H84 N O9 P	5,15	825,5852	78710	lipids	negative
PS(P-20:0/16:1(9Z))	C42 H80 N O9 P	4,27	773,5548	78803	lipids	positive
p-Salicylic acid	C7 H6 O3	1,31	138,0323	3263	metabolites	negative
Pseudouridine	C9 H12 N2 O6	0,68	244,0701	5734	metabolites	negative
PtdIns-(1,2-dihexanoyl)	C21 H39 O13 P	5,47	530,2124	64898	metabolites	positive

Pteroyltriglutamic acid	C29 H33 N9 O12	5,50	699,226	6381	lipids	positive
Pyridoxamine-5'-Phosphate	C8 H13 N2 O5 P	0,88	294,0617	236	metabolites	negative
Pyrroline hydroxycarboxylic acid	C5 H7 N O3	1,92	129,0422	6196	metabolites	positive
Pyruvic acid	C3 H4 O3	0,47	88,0164	117	metabolites	negative
Saccharin	C7 H5 N O3 S	0,84	229,0049	43328	metabolites	negative
ß-lactic acid	C3 H6 O3	0,54	90,0315	35392	metabolites	negative
Stearamide	C18 H37 N O	2,99	283,2869	34494	lipids	positive
Taurochenodeoxycholic acid	C26 H45 N O6 S	4,79	499,2964	57991	metabolites	negative
Taxiphyllin	C14 H17 N O7	0,67	311,1029	63629	metabolites	positive
Tenovin-6	C25 H34 N4 O2 S	0,30	454,2413	45456	lipids	positive
Testosterone sulfate	C19 H28 O5 S	4,16	368,1658	3558	metabolites	negative
Tetradecylamine	C14 H31 N	5,42	213,2457	3313	metabolites	positive
tetranor-PGDM	C16 H24 O7	7,99	328,1515	45422	metabolites	positive
TG(16:1(9Z)/20:4(5Z,8Z,11Z,14Z)/20:5(5Z,8Z,11Z,14Z,17Z))[iso6]	C59 H94 O6	10,26	898,7025	37447	lipids	positive
TG(17:0/20:0/22:4(7Z,10Z,13Z,16Z))[iso6]	C62 H112 O6	2,30	952,842	37715	metabolites	positive
TG(20:4(5Z,8Z,11Z,14Z)/20:5(5Z,8Z,11Z,14Z,17Z)/22:6(4Z,7Z,10Z,13Z,16Z,19Z))[iso6]	C65 H96 O6	10,20	972,7194	38905	lipids	positive
Thermozeaxanthin-13	C59 H90 O8	6,15	926,6679	71118	lipids	negative
THTC	C5 H8 O2 S	0,65	178,0303	68817	metabolites	negative
trans,trans-hepta-2,4,6-trienoic acid	C7 H8 O2	1,96	124,0523	45787	metabolites	negative
Tricosanamide	C41 H81 N O3	8,03	681,626	5896	lipids	negative
Tryptophan	C11 H12 N2 O2	1,30	204,0897	65534	metabolites	negative
Ubiquinone 8	C49 H74 O4	8,65	726,5553	4247	lipids	positive
Uric acid	C5 H4 N4 O3	0,60	168,0282	88	metabolites	positive
δ-Valerolactam	C5 H9 N O	1,15	99,0682	62467	metabolites	positive
